# TELO2-interacting protein 1 (TTI1), a novel Wnt/β-catenin target gene, decreases chemo-sensitivity in colorectal cancer by modulating DNA damage responses

**DOI:** 10.1186/s43556-026-00475-8

**Published:** 2026-06-12

**Authors:** Yuqiao Chen, Zheng Chen, Ya Wang, Yuanbing Yao, Youyu Zhang, Wentao Huang, Kun Song, Fengbo Tan, Fei Long, Changwei Lin, Qian Zhang, Wei Zhu, Wei Zhuang, Jianhua Zhou, Heli Liu, Shuai Xiao, Kai Fu

**Affiliations:** 1 Institute of Molecular Precision Medicine and Hunan Key Laboratory of Molecular Precision Medicine, Department of General Surgery, Xiangya Hospital, Central South University, Changsha, Hunan China; 2 Department of Thoracic Surgery, Xiangya Hospital, Central South University, Changsha, Hunan China; 3 Department of Neurosurgery, Xiangya Hospital, Central South University, Changsha, Hunan China; 4 Hengyang Medical School, The First Affiliated Hospital, Cancer Research Institute, University of South China, Hengyang, Hunan China; 5 Department of Gastrointestinal Surgery, Xiangya Hospital, Central South University, Changsha, Hunan China; 6 Department of Gastrointestinal Surgery, The Third Xiangya Hospital, Central South University, Changsha, Hunan China; 7 Department of Nutrition and Health, China Agricultural University, Beijing, China; 8 Department of Pathology, Xiangya Hospital, Central South University, Changsha, China; 9 MOE Key Lab of Rare Pediatric Diseases & Hunan Key Laboratory of Medical Genetics of the School of Life Sciences, Central South University, Changsha, Hunan China; 10 National Clinical Research Center for Geriatric Disorders, Changsha, Hunan China; 11 Hunan Key Laboratory of Aging Biology, Xiangya Hospital, Central South University, Changsha, Hunan China

**Keywords:** Colorectal Cancer, Wnt/β-catenin Signaling, DNA Damage Response, Chemo-sensitivity

## Abstract

Colorectal cancer is frequently driven by hyperactivation of Wnt/β-catenin signaling, which also contributes to reduced responsiveness to chemotherapy. However, how aberrant Wnt/β-catenin signaling enables colorectal cancer cells to tolerate chemotherapy-induced DNA damage remains elusive. Identifying actionable downstream effectors of this pathway may provide a more selective strategy to improve chemotherapy response while avoiding the toxicity associated with global Wnt inhibition. Here we show that *TELO2-interacting protein 1* (*TTI1*) is a direct transcriptional target of β-catenin/TCF in colorectal cancer. TTI1 expression correlates with β-catenin abundance and is elevated in tumors from patients with poor response to neoadjuvant chemotherapy. Mechanistically, TTI1 maintains the integrity of the TELO2-TTI1-TTI2 complex and stabilizes the DNA damage response kinases ATM and ATR. Genetic depletion of TTI1 destabilizes ATM and ATR, attenuates DNA damage signaling, impairs double-strand break repair, and sensitizes colorectal cancer cells to 5-fluorouracil and oxaliplatin. Conversely, restoration of TTI1 or ATM/ATR re-establishes DNA damage responses and chemo-insensitivity. Pharmacological suppression of the TTT complex by piperlongumine mimics TTI1 loss and enhances the anti-tumor activity of chemotherapy in cell lines, xenografts, *Apc*-mutant patient-derived organoids and *Apc*^min/+^ colonic adenomas. These findings define a β-catenin-TTI1-ATM/ATR axis that links Wnt/β-catenin activation to DNA damage tolerance and chemotherapy response in colorectal cancer. Targeting TTI1 is therefore a promising approach to improve chemotherapy efficacy in Wnt/β-catenin-activated colorectal cancer.

**Supplementary Information:**

The online version contains supplementary material available at 10.1186/s43556-026-00475-8.

## Introduction

Colorectal cancer (CRC) remains a leading cause of cancer mortality worldwide, and its burden continues to rise, particularly in developing countries [1]. For patients with advanced disease, systemic chemotherapy remains a central component of treatment, with 5-fluorouracil (5-FU)-based regimens forming the backbone of standard care [2, 3]. The addition of oxaliplatin (OXA) improves clinical response and is widely used in both neoadjuvant and advanced settings [4, 5]. However, therapeutic response remains heterogeneous, and a substantial proportion of patients show limited benefit from treatment. Because 5-FU and OXA exert their cytotoxic effects primarily through DNA damage, reduced chemotherapy response is thought to reflect enhanced tolerance to genotoxic stress and more efficient activation of DNA damage response pathways [6, 7].

Aberrant Wnt/β-catenin signaling is a defining molecular feature of CRC and is present in the vast majority of tumors, most commonly through mutations in *APC* or *CTNNB1* that drive β-catenin accumulation and transcriptional activation [8–10]. In addition to its established roles in intestinal homeostasis and tumor initiation, Wnt/β-catenin signaling has been implicated in reduced sensitivity to 5-FU-based chemotherapy [11, 12]. Pharmacological inhibition of this pathway can enhance chemotherapy response in preclinical models, but systemic Wnt blockade remains clinically challenging because of substantial on-target toxicity in normal tissues, particularly in the intestine, bone, and hematopoietic system [11–13]. These limitations suggest that therapeutically actionable downstream effectors of Wnt/β-catenin, rather than pathway-wide inhibition, may offer a more selective strategy to overcome chemo-insensitivity in CRC. However, the downstream mediators linking Wnt/β-catenin activation to chemotherapy tolerance remain poorly defined [14, 15].

The phosphatidylinositol-3 kinase-related kinase (PIKK) family coordinates cellular responses to genotoxic stress, with ATM and ATR acting as central sensors of DNA damage [16, 17]. Stability of these kinases depends on the TTT co-chaperone complex, composed of TELO2, TELO2-interacting protein 1 (TTI1), and TTI2 [18–20]. Within this complex, TTI1 functions as a central scaffold required for complex integrity and PIKK stabilization. Recent work has linked TTI1 to CRC progression and poor clinical outcome, supporting its relevance in colorectal tumor biology [21]. However, whether TTI1 is directly engaged by oncogenic Wnt/β-catenin signaling and whether it contributes to chemotherapy tolerance through ATM/ATR-dependent DNA damage control remain unknown.

Here we identify *TTI1* as a direct transcriptional target of Wnt/β-catenin signaling in CRC. We show that *TTI1* is elevated in tumors with poor response to neoadjuvant chemotherapy and is required to maintain ATM and ATR stability, sustain DNA damage signaling, and support double-strand break repair following chemotherapy. Genetic depletion of *TTI1* impairs DNA damage repair and sensitizes CRC cells to 5-FU and OXA. To test the therapeutic potential of this pathway, we further used piperlongumine (PL), a small molecule reported to disrupt RUVBL1/2-TTT complex assembly and destabilize TTT components [22, 23]. PL impaired DNA damage repair and enhanced chemotherapy response in CRC models in vitro and in vivo. Overall, our findings reveal a Wnt/β-catenin-TTI1-ATM/ATR signaling axis that drives chemotherapy resistance, suggesting that targeting TTI1 could be an effective strategy to treat Wnt-activated colorectal cancer.

## Results

### TTI1 is upregulated in CRC tissues and is associated with poor response to chemotherapy

**Fig. 1:**
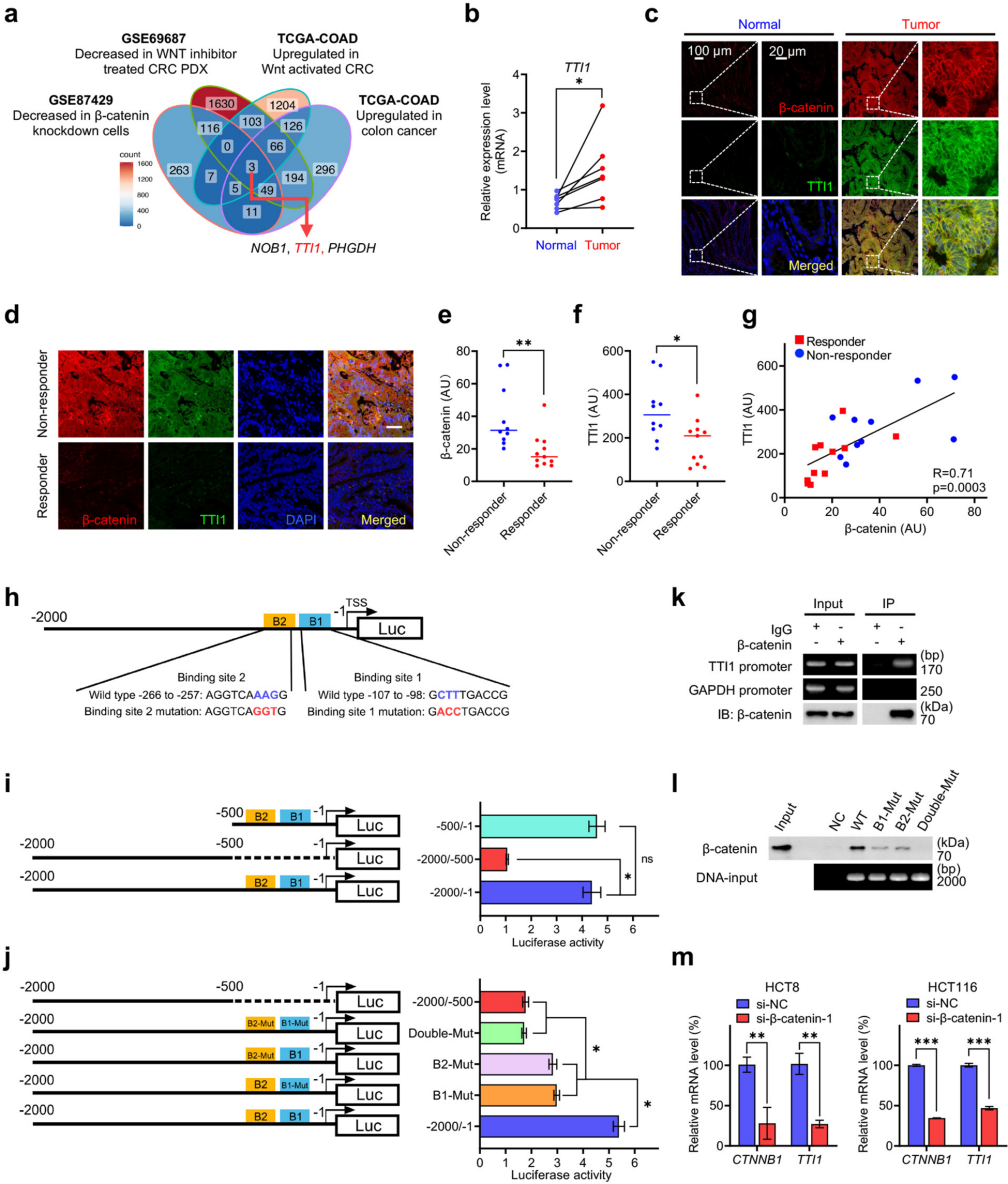
TTI1 is a novel Wnt/β-catenin downstream target gene that reduces sensitivity to chemotherapeutic drugs in colorectal cancer.** a** A comprehensive analysis of multiple transcriptomic datasets, including GSE87429, GSE69687, and TCGA-COAD, identified TTI1 as a candidate downstream target gene of Wnt/β-catenin signaling. **b** TTI1 mRNA expression levels in tumor tissues and paired normal tissues from CRC patients in our institution were compared using a paired t-test. **c** Protein levels of β-catenin and TTI1 in tumors from colorectal cancer (CRC) patients and paired normal tissues were determined by immunohistochemistry. **d** Representative immunofluorescence images of TTI1 and β-catenin in colorectal tumor sections from chemotherapy responders and non-responders, as determined by tumor regression grade (TRG) after 5-FU-based neoadjuvant chemotherapy. Scale bar, 50 μm. **e**, **f** Quantification of grayscale values for TTI1 and β-catenin in chemotherapy responders and non-responders shown in (**d**). **g** Linear regression analysis of TTI1 and β-catenin protein levels in tumor tissues from CRC patients (blue, chemotherapy non-responders; red, chemotherapy responders). **h** Schematic representation of putative TCF/LEF-binding elements located at −107/−98 bp and −266/−257 bp upstream of the transcription start site (TSS) of TTI1. **i**, **j** Schematic diagrams of the *TTI1* promoter constructs and their derivatives (−2000/−1, −500/−1, −2000/−500, B1-Mut, B2-Mut, and Double-Mut) are shown on the left. Luciferase activities of the indicated *TTI1* promoter constructs and derivatives were measured in 293 T cells (right). The pRL-TK Renilla luciferase reporter was co-transfected into each sample to normalize transfection efficiency. Reporter luciferase activity was normalized to Renilla luciferase activity. Data are shown as mean ± SEM from three independent experiments, each performed in triplicate. **k** Chromatin immunoprecipitation (ChIP) assays showed that β-catenin binds to the *TTI1* promoter region. PCR analyses of nuclear extracts (input, left) and chromatin immunoprecipitates obtained with IgG (negative control) or a β-catenin monoclonal antibody (IP, right) are shown. The *GAPDH* promoter served as an additional negative control. Inputs and chromatin immunoprecipitates were immunoblotted for β-catenin (bottom). **l** Biotin-labeled oligonucleotides corresponding to the *TTI1* promoter constructs (WT, B1-Mut, B2-Mut, and Double-Mut) were incubated with cell lysates and streptavidin magnetic beads. The precipitated proteins were immunoblotted for β-catenin. **m** *CTNNB1* and *TTI1* mRNA levels were measured by RT-qPCR after transfection with nonspecific control siRNA (si-NC) or *CTNNB1*-specific siRNA (si-β-catenin-1). AU, arbitrary unit. Statistical significance was determined by two-tailed Student’s t-test or one-way ANOVA. ns, *p* > 0.05; *, *p* < 0.05; **, *p* < 0.01; ***, *p* < 0.001

Aberrant activation of Wnt/β-catenin signaling is a hallmark of colorectal cancer (CRC) [9], with cytoplasmic accumulation of β-catenin observed in over 80% of cases, enabling its nuclear translocation and transcriptional activation of downstream target genes [24, 25]. A comprehensive analysis of multiple transcriptomics datasets indicated that *TTI1*, *NOB1*, and *PHGDH* were novel candidate downstream genes of the β-catenin-dependent Wnt pathway (Fig. 1a). Among these candidates, TTI1 was the only gene significantly downregulated after β-catenin silencing in CRC cells (Additional file 1: Fig. S1a) and was therefore selected for further investigation. Consistently, *TTI1* expression was suppressed after knockdown of β-catenin in vitro (Additional file 1: Fig. S1b), and after treatment with the Wnt ligand secretion inhibitor ETC159 in vivo (Additional file 1: Fig. S1c). Besides, *TTI1* expression was elevated in cancer tissues compared to paracancerous tissues (Additional file 1: Fig. S1d), with notably higher levels observed in cancer tissues exhibiting high Wnt/β-catenin pathway activity compared to those with low activity (Additional file 1: Fig. S1e).

Elevated TTI1 transcript and protein levels in tumor tissues from CRC patients were further confirmed by RT-qPCR and immunohistochemistry, respectively, compared with normal tissues (Fig. 1b-c). The main clinical characteristics of these patients are listed in Additional file 2: Table S1. Capecitabine, an oral precursor to 5-FU, plus OXA or 5-FU plus OXA are common neoadjuvant chemotherapy strategies for treatment of CRC [26]. To determine whether TTI1 and β-catenin are associated with response to chemotherapy in CRC, we detected their expression levels in patients classified according to the American Joint Committee on Cancer (AJCC) tumor regression grade (TRG) after 5-FU-based neoadjuvant chemotherapy, including chemo-responders (AJCC TRG 0–1) and chemo-non-responders (AJCC TRG 3) [27]. The results showed that both β-catenin and TTI1 were more highly expressed in non-responders than in responders (Fig. 1d-f). Furthermore, the elevated TTI1 levels positively correlated with increased β-catenin levels in human CRC tissues (Fig. 1g). The main clinicopathological items of patients with different chemotherapy response are listed in Additional file 3: Table S2. Together, these data suggest that aberrant β-catenin accumulation activates TTI1 expression through β-catenin-dependent Wnt signaling, thereby contributing to reduced responsiveness of CRC to chemotherapeutic drugs.

### TTI1 is a direct transcriptional target of Wnt/β-catenin signaling

To determine whether *TTI1* is directly regulated by β-catenin/TCF, we examined the *TTI1* genomic region for potential TCF/LEF-binding sites (WWCAAWG, W = A/T). PROMO analysis [28] identified two putative TCF/LEF-binding sites located at −107/−98 bp and −266/−257 bp upstream of the *TTI1* transcription start site (TSS) (Fig. 1h). We therefore generated luciferase reporter constructs containing the *TTI1* promoter or its derivatives, including −2000/−1, −500/−1, −2000/−500, binding site 1 mutant (B1-Mut), binding site 2 mutant (B2-Mut), and double mutant (Double-Mut). Luciferase activity was markedly reduced in cells transfected with the −2000/−500 construct compared with those transfected with the −2000/−1 or −500/−1 constructs, indicating that the Wnt-responsive region is located within 500 bp upstream of the *TTI1* TSS (Fig. 1i). Mutation of either predicted TCF/LEF-binding site reduced luciferase activity, while simultaneous mutation of both sites decreased reporter activity to a level comparable to that of the −2000/−500 construct (Fig. 1j). Consistently, ChIP assays showed β-catenin enrichment at the *TTI1* promoter (Fig. 1k). DNA pull-down assays further showed that biotin-labeled *TTI1* promoter fragments precipitated β-catenin, whereas mutation of either TCF/LEF-binding site reduced β-catenin binding and mutation of both sites markedly diminished this interaction (Fig. 1l). In Wnt/β-catenin-activated CRC cells, β-catenin accumulation was accompanied by increased TTI1 expression, in contrast to RKO cells with intact Wnt/β-catenin signaling (Additional file 1: Fig. S2a-b). Moreover, β-catenin knockdown significantly suppressed *TTI1* mRNA expression in both HCT8 cells, which harbor loss-of-function *APC* mutation, and HCT116 cells, which harbor stabilized β-catenin due to a Ser45 mutation (Fig. 1m). Re-expression of siRNA-resistant β-catenin(M) in β-catenin-depleted HCT8 cells restored *TTI1* mRNA expression (Additional file 1: Fig. S3). Together, these findings demonstrate that β-catenin directly binds to the −107/−98 bp and −266/−257 bp regions of the *TTI1* promoter and activates *TTI1* transcription.

### TTI1 is required for the stability of ATM and ATR proteins

**Fig. 2:**
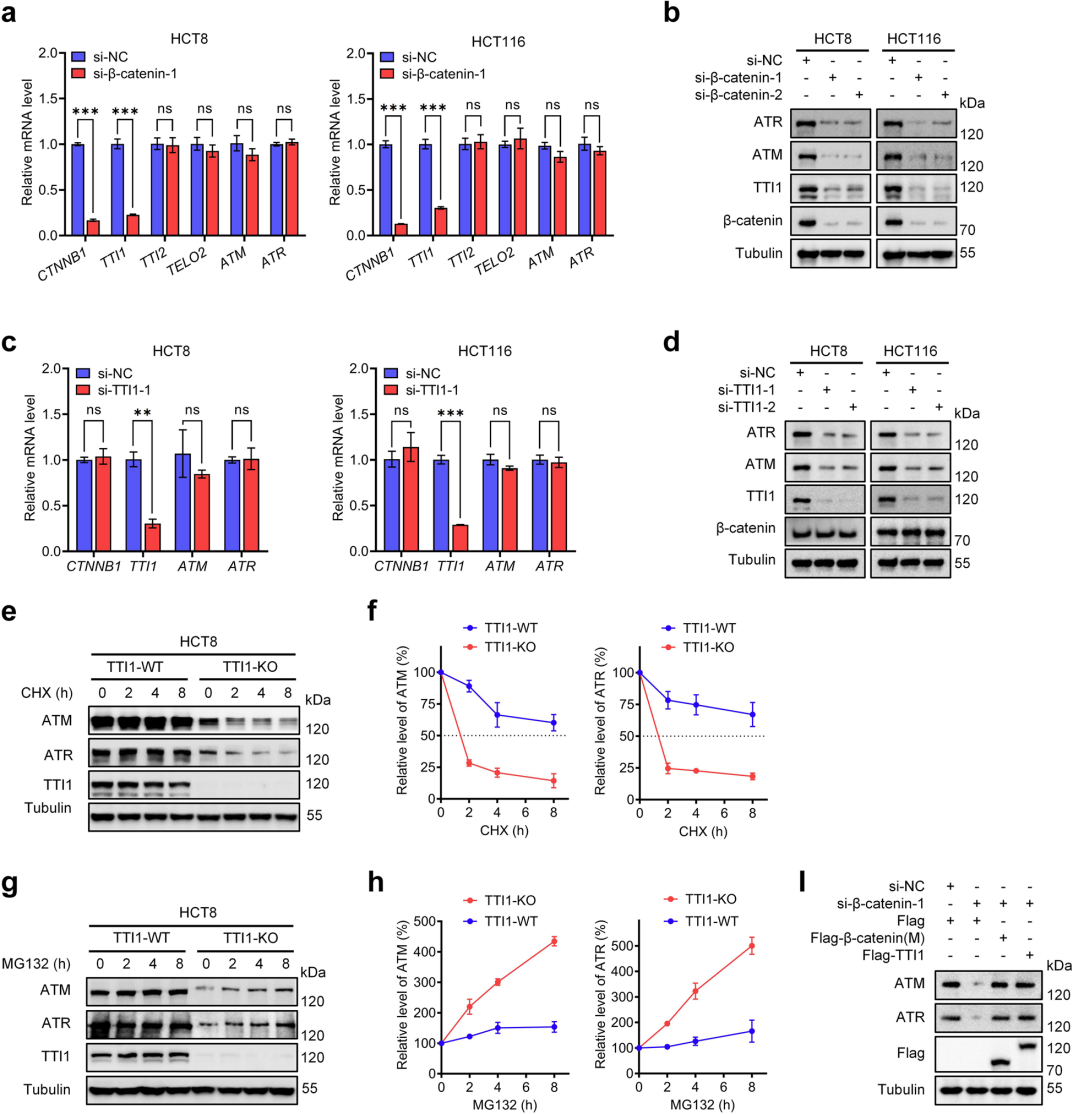
β-catenin-regulated TTI1 maintains ATM and ATR protein stability in CRC cells.** a**, **b** HCT8 and HCT116 cells were transfected with nonspecific control siRNA (si-NC), β-catenin-specific siRNA (si-β-catenin-1) for RT-qPCR analysis (**a**), or two independent β-catenin-targeting siRNAs for immunoblotting (**b**) for 72 h. The mRNA levels of the indicated genes were measured by RT-qPCR, and the expression levels of the indicated proteins were detected by immunoblotting, with Tubulin as a loading control. **c**, **d** HCT8 and HCT116 cells were transfected with si-NC, TTI1-specific siRNA (si-TTI1-1) for RT-qPCR analysis (**c**), or two independent TTI1-targeting siRNAs for immunoblotting (**d**) for 72 h. The mRNA levels of the indicated genes were measured by RT-qPCR, and the expression levels of the indicated proteins were detected by immunoblotting, with Tubulin as a loading control. **e**, **g** The dynamics of ATM and ATR protein expression were examined in HCT8 TTI1-wild-type (TTI1-WT) and TTI1-knockout (TTI1-KO) cells treated with cycloheximide (CHX) (**e**) or the proteasome inhibitor MG132 (**g**) for the indicated times. Whole-cell lysates were subjected to immunoblotting for the indicated proteins, with Tubulin as a loading control. **f** Quantification of ATM and ATR protein levels in (**e**), shown as percentages relative to the 0 h time point. **h** Quantification of ATM and ATR protein levels in (**g**), shown as percentages relative to the 0 h time point. **i** HCT8 cells were transfected with si-NC or si-β-catenin-1 for 36 h and then transfected with the indicated plasmids, including empty vector (Flag), siRNA-resistant Flag-β-catenin(M), or Flag-TTI1, for another 36 h. Whole-cell lysates were analyzed by immunoblotting for the indicated proteins. Data in (**a**), (**c**), (**f**), and (**h**) are presented as the mean ± SEM of three independent experiments. Statistical significance in (**a**) and (**c**) was determined by two-tailed Student’s t-test. ns, *p* > 0.05; **, *p* < 0.01; ***, *p* < 0.001

TTI1, TTI2, and TELO2 constitute the TTT complex, which is essential for the stability of ATM and ATR proteins [19]. To explore whether β-catenin influences the expression of ATM and ATR at the mRNA and protein levels, RT-qPCR and immunoblotting were performed after β-catenin knockdown in HCT8 and HCT116 cells. β-catenin depletion markedly reduced TTI1 mRNA and protein levels, but did not decrease the mRNA expression of ATM, ATR, TTI2, or TELO2 (Fig. 2a-b; Additional file 1: Fig. S4a). In contrast, ATM and ATR protein levels were markedly reduced in β-catenin-depleted cells (Fig. 2b; Additional file 1: Fig. S4a). Similar results were observed after TTI1 knockdown, which substantially decreased ATM and ATR protein abundance without affecting their mRNA expression (Fig. 2c-d; Additional file 1: Fig. S4b). Importantly, re-expression of siRNA-resistant β-catenin(M) or TTI1 restored ATM and ATR protein levels in β-catenin-depleted HCT8 cells (Fig. 2i), supporting TTI1 as a downstream effector of β-catenin in maintaining ATM/ATR abundance. We next generated TTI1-knockout HCT8 cells using the CRISPR-Cas9 system. ATM and ATR proteins decayed more rapidly after cycloheximide (CHX) treatment in TTI1-knockout cells than in TTI1-wild-type cells (Fig. 2e-f). Moreover, treatment with the proteasome inhibitor MG132 rapidly restored ATM and ATR protein levels in TTI1-deficient cells (Fig. 2g-h). Collectively, these findings indicate that β-catenin-regulated TTI1 maintains ATM and ATR protein stability in CRC cells.

### TTI1 deficiency dampens DNA double-strand break (DSB) repair

**Fig. 3:**
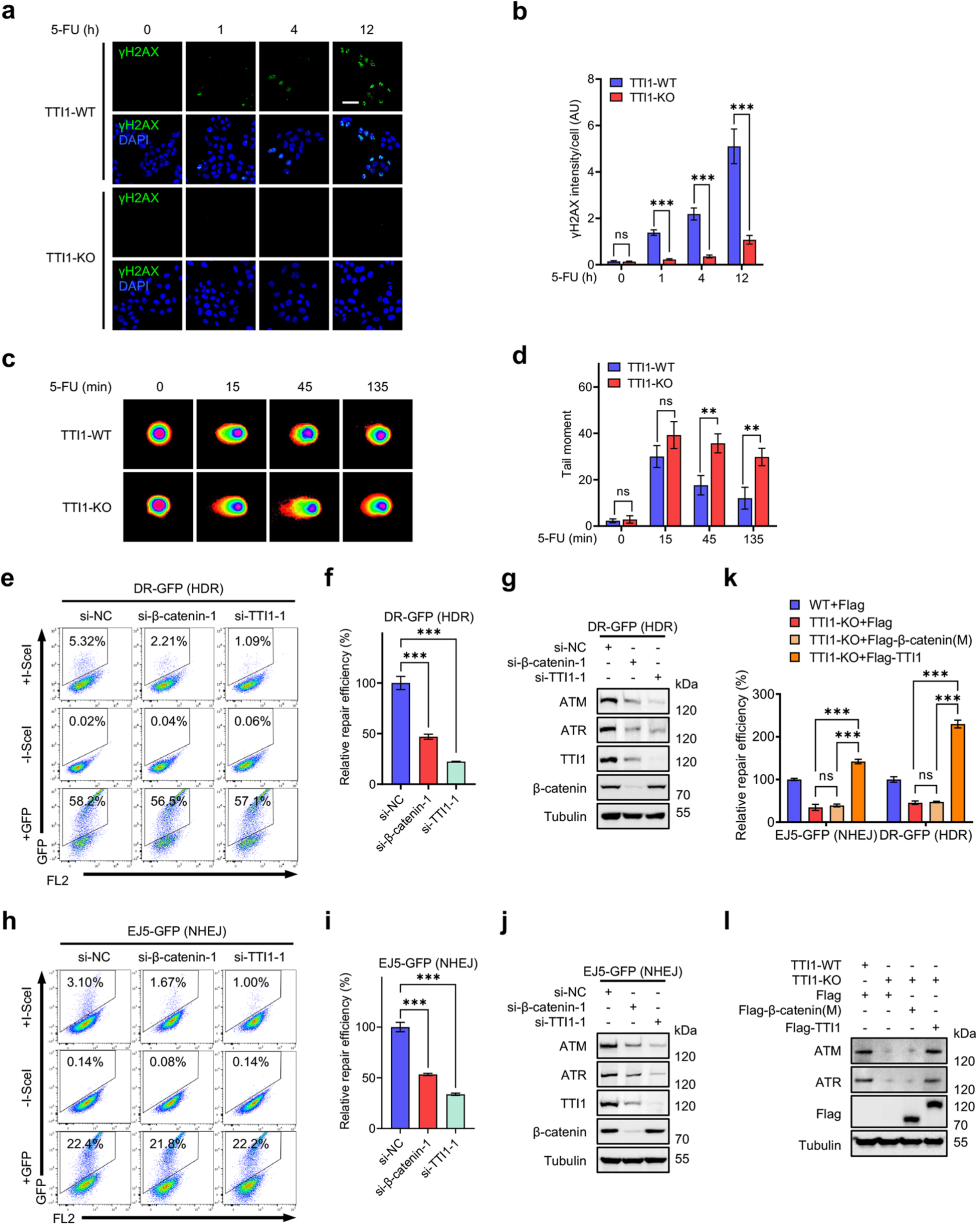
TTI1 is required for repairing chemotherapeutic agents-induced DSBs.** a** Immunofluorescence images of γH2AX in TTI1-WT and TTI1-KO HCT8 cells collected at the indicated time points following treatment with 10 µM 5-fluorouracil (5-FU), with nuclei counterstained with DAPI. Scale bar, 50 μm. **b** Quantification of γH2AX foci grayscale values per cell in (**a**). Ten to twenty random fields of view were analyzed at each time point. **c** Representative images of neutral comet assays in TTI1-WT and TTI1-KO HCT8 cells at the indicated time points after treatment with 40 µM 5-FU. **d** Quantification of tail moments (TM values) in cells shown in (**c**). TM values were measured in 10–30 single cells from 10 random fields of view at each time point. **e**, **h** Flow cytometric analysis of the effect of TTI1 knockdown on DNA damage repair efficiency. Twenty-four hours after transfection with si-NC or si-TTI1, U2OS cells were co-transfected with the HDR or NHEJ reporter together with I-SceI to induce DSBs at unique I-SceI sites. Representative flow cytometry plots showing the frequency of GFP-positive cells 48 h after transfection are shown, indicating HDR (**e**) or NHEJ (**h**) efficiency. Cells transfected with GFP were used to indicate transfection efficiency, whereas cells transfected with the HDR or NHEJ reporter alone served as blank controls. **f**, **i** Quantification of relative HDR (**f**) or NHEJ (**i**) efficiency from three independent cell cultures. **g**, **j** Representative immunoblots of the indicated proteins in U2OS cells transfected as described in (**e**) and (**h**), respectively. **k** Quantification of HDR and NHEJ repair efficiency in TTI1-WT or TTI1-KO HCT8 cells transfected with Flag, siRNA-resistant Flag-β-catenin(M), or Flag-TTI1, summarized from three independent experiments. Representative flow cytometry plots are shown in Additional file 1: Fig. S6. **l** Whole-cell lysates from HCT8 cells treated as described in (**k**) were subjected to immunoblotting for the indicated proteins, with Tubulin as a loading control. Statistical significance was determined by two-tailed Student’s t-test or one-way ANOVA. ns, *p* > 0.05; **, *p* < 0.01; ***, *p* < 0.001

Chemotherapeutic agents induce DNA double-strand breaks (DSBs), a highly toxic form of DNA damage [29]. We first examined γH2AX foci, a sensitive marker of DSBs, to assess whether TTI1 affects DSB repair in CRC cells [30]. 5-FU robustly induced γH2AX foci in TTI1-WT HCT8 cells, whereas this response was markedly attenuated in TTI1-KO cells (Fig. 3a-b). Neutral comet assays showed comparable initial DNA damage in TTI1-WT and TTI1-KO cells shortly after 5-FU treatment. However, comet tail moments gradually decreased in TTI1-WT cells, but remained elevated in TTI1-KO cells, indicating impaired DSB repair upon TTI1 loss (Fig. 3c-d).

Homology-directed repair (HDR) and non-homologous end joining (NHEJ) have been recognized as the major pathways for repairing DSBs in eukaryotic cells [31]. We next evaluated the role of TTI1 in DSB repair pathways in cancer cells. U2OS cells, an osteosarcoma cell line with intact ATM and ATR signaling pathways, were selected because they are widely used in DNA repair studies and exhibit robust DNA damage response. HDR and NHEJ reporter systems, which include recognition sites for the rare-cutting endonuclease I-SceI that can induce double-strand breaks at specific locations, were used to evaluate repair efficiency. We found that β-catenin or TTI1 knockdown markedly reduced HDR and NHEJ repair efficiencies compared with control cells (Fig. 3e-j). Immunoblotting confirmed efficient depletion of β-catenin and TTI1 under these conditions (Fig. 3g and j). Importantly, re-expression of TTI1, but not siRNA-resistant β-catenin(M), restored HDR and NHEJ repair efficiency in TTI1-KO HCT8 cells (Fig. 3k; Additional file 1: Fig. S6). Consistently, TTI1 re-expression restored ATM and ATR protein levels in TTI1-KO cells, whereas β-catenin(M) did not (Fig. 3l). Together, these findings indicate that TTI1 acts as a functional downstream effector of β-catenin to maintain ATM/ATR abundance and support DSB repair.

### Loss of TTI1 attenuates the DNA damage repair signaling and sensitizes CRC cells to chemotherapy

**Fig. 4:**
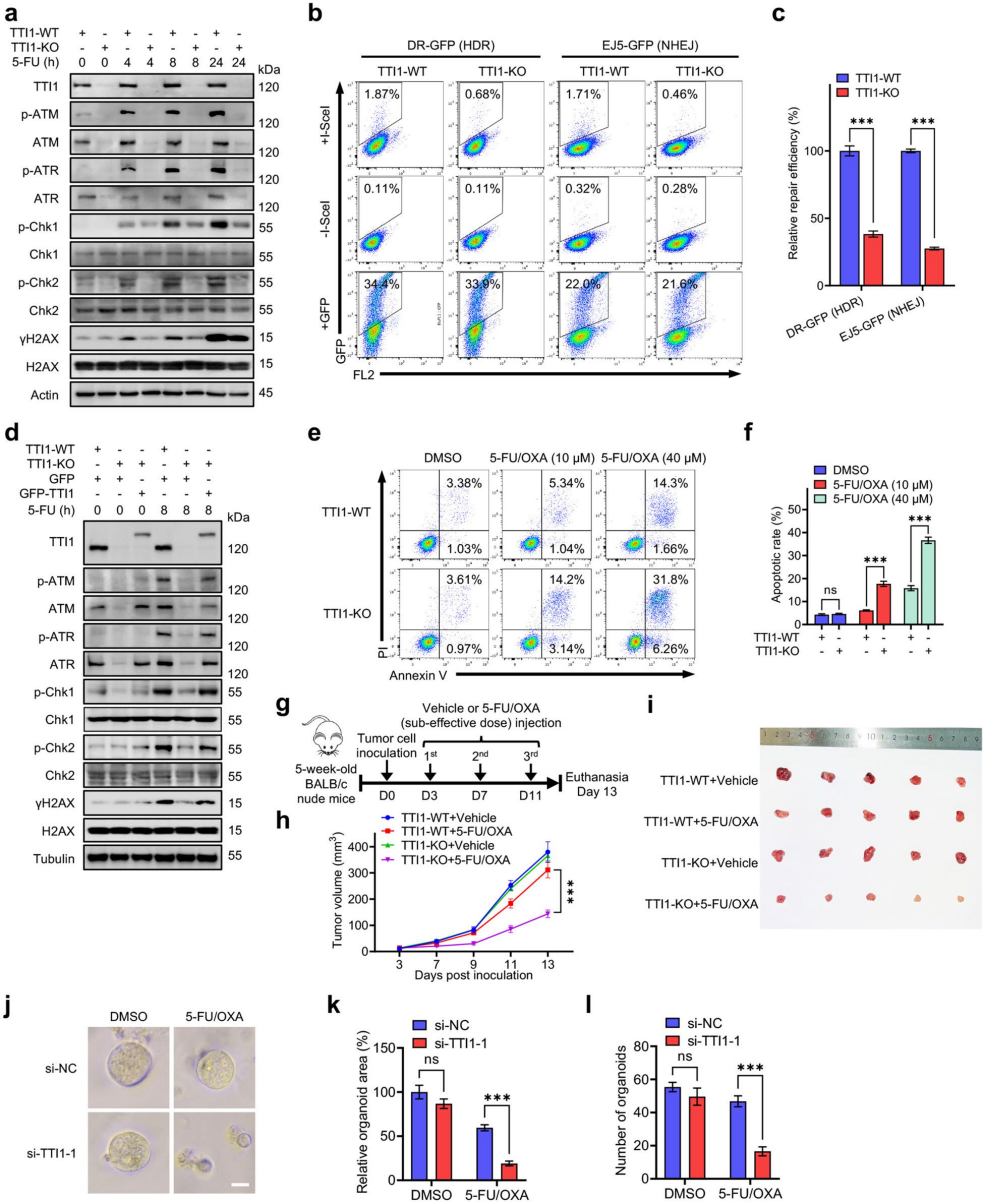
TTI1 depletion dampens the DNA damage repair signaling cascade and sensitizes CRC cells to chemotherapeutic drugs.** a** Whole-cell lysates from TTI1-WT and TTI1-KO HCT8 cells were collected at the indicated time points after treatment with 10 μM 5-FU and immunoblotted for the indicated proteins, with β-actin as a loading control. **b** Flow cytometric analysis of the effect of TTI1 deletion on DNA damage repair efficiency. TTI1-WT and TTI1-KO HCT8 cells were co-transfected with the HDR or NHEJ reporter together with I-SceI for 48 h. Representative flow cytometry plots showing the frequency of GFP-positive cells are shown, indicating HDR or NHEJ efficiency. Cells transfected with GFP were used to indicate transfection efficiency, whereas cells transfected with the HDR or NHEJ reporter alone served as blank controls. **c** Quantification of relative HDR or NHEJ efficiency in (**b**), summarized from three independent cell cultures. **d** TTI1-WT HCT8 cells were transduced with lentiviral vectors carrying GFP, and TTI1-KO HCT8 cells were transduced with lentiviral vectors carrying either GFP or GFP-TTI1. Whole-cell lysates were collected at the indicated time points after treatment with 10 μM 5-FU and immunoblotted for the indicated proteins. **e** TTI1-WT and TTI1-KO HCT8 cells were treated with the indicated concentrations of 5-FU and OXA for 24 h and then stained with propidium iodide (PI)/Annexin V. Representative flow cytometry plots of apoptotic cells are shown. **f** Quantification of apoptotic cell rates (PI^−^/Annexin V^+^ and PI^+^/Annexin V^+^) in (**e**). **g** Schematic of the experimental timeline for the subcutaneous xenograft assay. **h** Tumor growth curves of mice subcutaneously injected with TTI1-WT or TTI1-KO HCT8 cells and treated by intraperitoneal administration of a sub-effective dose of chemotherapy (2.5 mg/kg 5-FU and 2.5 mg/kg OXA) or vehicle control. **i** Representative images of tumors dissected from mice on day 13 after implantation. **j** Patient-derived organoids were transfected with si-NC or si-TTI1-1 for 48 h and then treated with DMSO or 1 μM 5-FU plus OXA for another 72 h. Representative images of patient-derived organoids in each indicated group are shown. Scale bar, 50 μm. **k**, **l** Quantification of organoid size (**k**) and number (**l**) in each indicated group shown in (**j**). In (**k**), organoid size was normalized to the si-NC + DMSO group (set to 100%). Data were summarized from 10 individual organoids or 5 random fields in each indicated group. Statistical significance was determined by two-tailed Student’s t-test or one-way ANOVA. ns, *p* > 0.05; ***, *p* < 0.001

ATM and ATR are central regulators of DNA damage signaling and activate downstream effectors such as Chk1, Chk2 and H2AX [32, 33]. We therefore examined whether TTI1 depletion affects this signaling cascade in CRC cells. Phosphorylation of Chk1, Chk2, and H2AX was robustly induced by both 5-FU and OXA in TTI1-sufficient HCT8 cells, whereas these responses were markedly attenuated in TTI1-deficient cells, which showed reduced ATM and ATR protein levels (Fig. 4a; Additional file 1: Fig. S5a). Consistently, HDR and NHEJ repair efficiencies were significantly reduced in TTI1-deficient cells compared with TTI1-sufficient cells (Fig. 4b-c). Reconstitution of TTI1 in TTI1-deficient HCT8 cells restored ATM and ATR expression and reactivated ATM/ATR-mediated signaling cascades (Figs. 4d; 3l). Accordingly, ectopic expression of TTI1 restored the reduced sensitivity of TTI1-KO HCT8 cells to both 5-FU and OXA (Additional file 1: Fig. S5b-c). Moreover, ectopic expression of ATM and ATR rescued the activation of DNA damage response signaling in both β-catenin- and TTI1-deficient CRC cells (Additional file 1: Fig. S7a). Functionally, ectopic expression of ATM and ATR, but not β-catenin, restored chemo-insensitivity in TTI1-deficient CRC cells (Additional file 1: Fig. S7b-d), supporting that TTI1 acts downstream of Wnt/β-catenin signaling to maintain ATM/ATR-mediated DNA damage responses.

Deletion of TTI1 significantly increased apoptosis induced by 5-FU and OXA in Wnt/β-catenin-activated CRC cells, as shown by propidium iodide (PI)/Annexin V staining (Fig. 4e-f). Conversely, ectopic expression of TTI1 substantially reduced the sensitivity of RKO cells to chemotherapeutic agents (Additional file 1: Fig. S2c-e), further supporting the role of TTI1 in CRC chemo-insensitivity. To assess this effect in vivo, we performed subcutaneous xenograft assays using TTI1-WT and TTI1-KO HCT8 cells in BALB/c nude mice. Four days after tumor cell injection, mice were treated with vehicle or a sub-effective dose of 5-FU plus OXA every four days (2.5 mg/kg 5-FU and 2.5 mg/kg OXA; Fig. 4g). Compared with TTI1-WT tumors, TTI1-KO tumors were markedly more sensitive to 5-FU/OXA treatment, as indicated by significantly reduced tumor growth (Fig. 4h-i). TUNEL staining and cleaved-caspase-3 immunostaining further showed extensive apoptosis in 5-FU/OXA-treated TTI1-KO tumors, whereas only minimal apoptosis was observed in similarly treated TTI1-WT tumors (Additional file 1: Fig. S8a-d). These findings indicate that TTI1 loss enhances tumor sensitivity to chemotherapeutic agents.

Activating alterations in the Wnt/β-catenin pathway are highly prevalent in CRC, most commonly due to *APC* mutations, which lead to β-catenin accumulation and downstream transcriptional activation [34]. To evaluate the role of TTI1 in CRC with high Wnt/β-catenin activity, we established patient-derived CRC organoids from a treatment-naive patient harboring an *APC* mutation (c.3922A > T, p.K1308*). TTI1 knockdown alone had no significant effect on organoid growth, but markedly sensitized the organoids to 5-FU and OXA, as reflected by reduced organoid size and number (Fig. 4j-l). Moreover, consistent with a recent report showing low TTI1 expression in normal colorectal tissues but elevated expression in colorectal cancer [21], TTI1 expression was minimal in the normal colonic epithelial cell line NCM460 but markedly higher in HCT8 and HCT116 cells (Additional file 1: Fig. S9a). Consistently, TTI1 depletion substantially enhanced chemo-sensitivity in HCT8 and HCT116 cells, while exerting only a minimal effect in NCM460 cells under the same treatment conditions (Additional file 1: Fig. S9b-c). Together, these results demonstrate that TTI1 is essential for reducing the sensitivity of CRC cells to chemotherapeutic agents.

### PL treatment impairs DNA damage response and enhances the efficacy of chemotherapeutic drugs

**Fig. 5:**
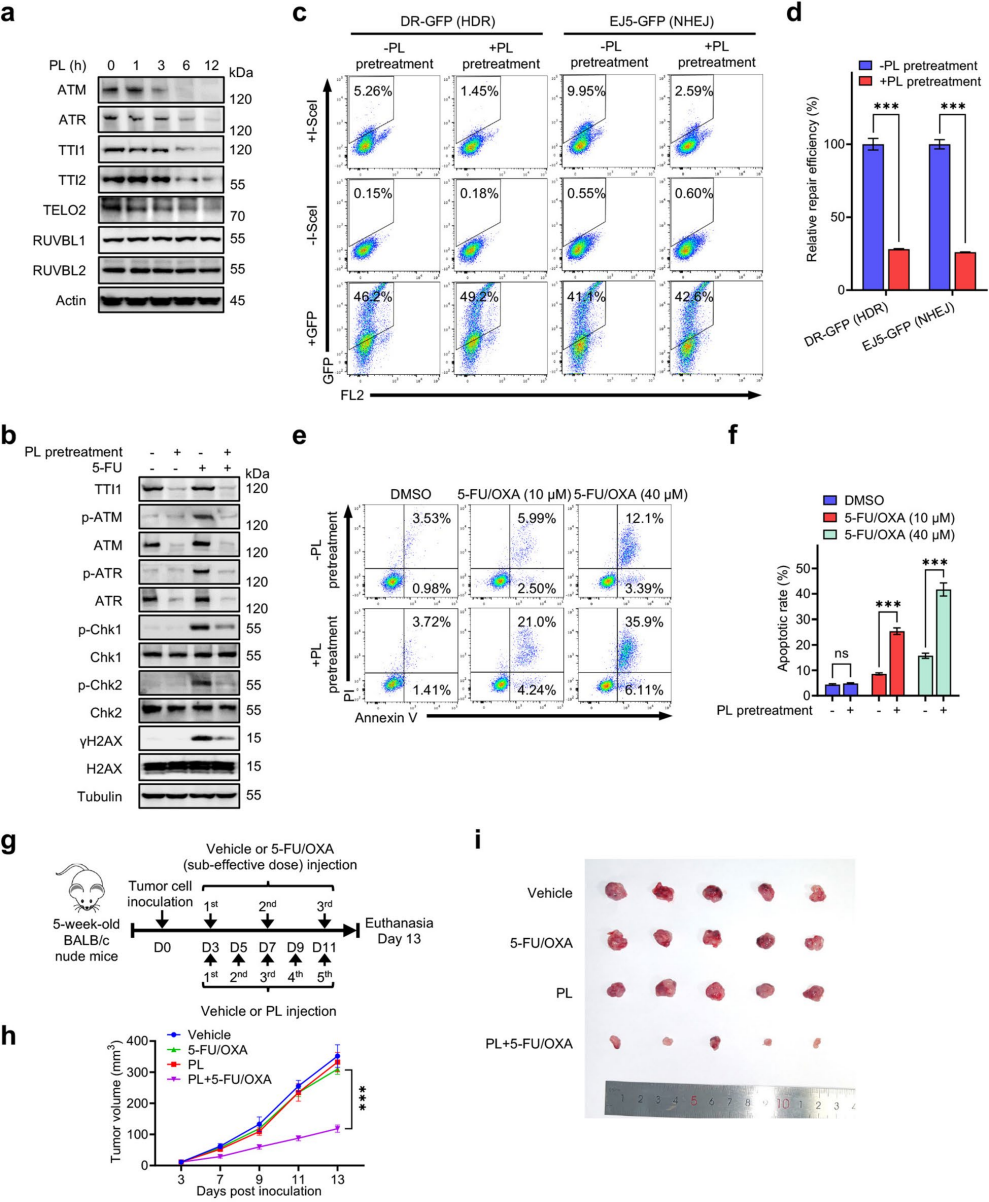
PL treatment attenuates chemotherapeutic agents-induced DNA damage responses in CRC cells.** a** HCT8 cells were pretreated with 2 µM piperlongumine (PL) for the indicated time periods and then immunoblotted for the indicated proteins. **b** HCT8 cells were pretreated with or without 2 µM PL for 8 h and then treated with 10 µM 5-FU for another 12 h. Protein levels of the indicated targets were then examined by immunoblotting. **c** Flow cytometric analysis of the effect of PL pretreatment on DNA damage repair efficiency. HCT8 cells were pretreated with or without 2 µM PL for 24 h and then co-transfected with the HDR or NHEJ reporter together with I-SceI. Representative flow cytometry plots showing the frequency of GFP-positive cells 48 h after transfection are shown, indicating HDR or NHEJ efficiency. Cells transfected with GFP were used to indicate transfection efficiency, whereas cells transfected with the HDR or NHEJ reporter alone served as blank controls. **d** Quantification of relative HDR or NHEJ efficiency in (**c**), summarized from three independent cell cultures. **e** HCT8 cells were pretreated with or without 2 µM PL for 24 h and then treated with the indicated doses of chemotherapeutic agents, followed by PI/Annexin V staining and flow cytometric analysis. Representative flow cytometry plots of apoptotic cells are shown. **f** Quantification of apoptotic cell rates in (**e**). **g** Schematic of the experimental timeline for the subcutaneous xenograft assay. **h** HCT8 xenograft growth curves following intraperitoneal administration of PL (5 mg/kg) alone, a sub-effective dose of chemotherapy alone (2.5 mg/kg 5-FU and 2.5 mg/kg OXA), PL (5 mg/kg) plus chemotherapy (2.5 mg/kg 5-FU and 2.5 mg/kg OXA), or vehicle control. **i** Representative images of tumors dissected from mice on day 13 after HCT8 cell implantation, as shown in (**g**) and (**h**). Statistical significance was determined by two-tailed Student’s t-test or one-way ANOVA. ns, *p* > 0.05; ***, *p* < 0.001

A recent study reported that PL directly binds RuvB Like AAA ATPase 1/2 (RUVBL1/2), dissociates them from the TTT complex, and thereby destabilizes TTI1, TTI2, and TELO2 [23]. Consistently, PL treatment decreased the protein levels of all TTT components and ATM/ATR in a time-dependent manner, while RUVBL1/2 protein levels remained largely unchanged (Fig. 5a). PL also reduced the association of TELO2 with TTI1 and TTI2, whereas TTI1 deficiency abolished the TELO2-TTI2 interaction, indicating disruption of the TTT complex (Additional file 1: Fig. S10). Similar reductions in TTT components and ATM/ATR were observed in CRISPR/Cas9-mediated TTI1-knockout CRC cells (Additional file 1: Fig. S11). We next examined whether PL impairs DNA damage repair signaling. PL pretreatment markedly attenuated 5-FU-induced activation of ATM, ATR, Chk1, Chk2, and H2AX (Fig. 5b). Consistently, 5-FU-induced γH2AX foci formation was reduced in HCT8 cells pretreated with PL compared with cells without PL pretreatment (Additional file 1: Fig. S12a-b). HDR and NHEJ reporter assays further showed that PL pretreatment impaired both DSB repair pathways in HCT8 cells (Fig. 5c-d). Functionally, PL alone caused little to no increase in apoptosis, whereas PL pretreatment substantially enhanced 5-FU/OXA-induced apoptosis and reduced cell viability in HCT8 cells (Fig. 5e-f; Additional file 1: Fig. S14a). Moreover, re-expression of TTI1 attenuated the inhibitory effect of PL plus 5-FU/OXA on clonogenic survival in both HCT8 and HCT116 cells (Additional file 1: Fig. S13), supporting that the chemosensitizing effect of PL is at least partly mediated through TTI1 suppression. To evaluate whether PL enhances chemosensitivity in vivo, mice bearing HCT8 xenografts were treated with a sub-effective dose of 5-FU/OXA every four days, while PL was administered intraperitoneally every two days. The combination of PL with 5-FU/OXA markedly suppressed tumor growth compared with chemotherapy alone (Fig. 5h-i). In situ TUNEL assays further showed a higher percentage of apoptotic cells in the PL plus 5-FU/OXA group than in the chemotherapy-alone group (Additional file 1: Fig. S14b-c). Together, these results indicate that PL impairs TTT complex-dependent DNA damage responses and enhances the anti-tumor effect of chemotherapeutic drugs.

### PL sensitizes CRC with hyperactivated Wnt/β-catenin signaling to chemotherapy

**Fig. 6:**
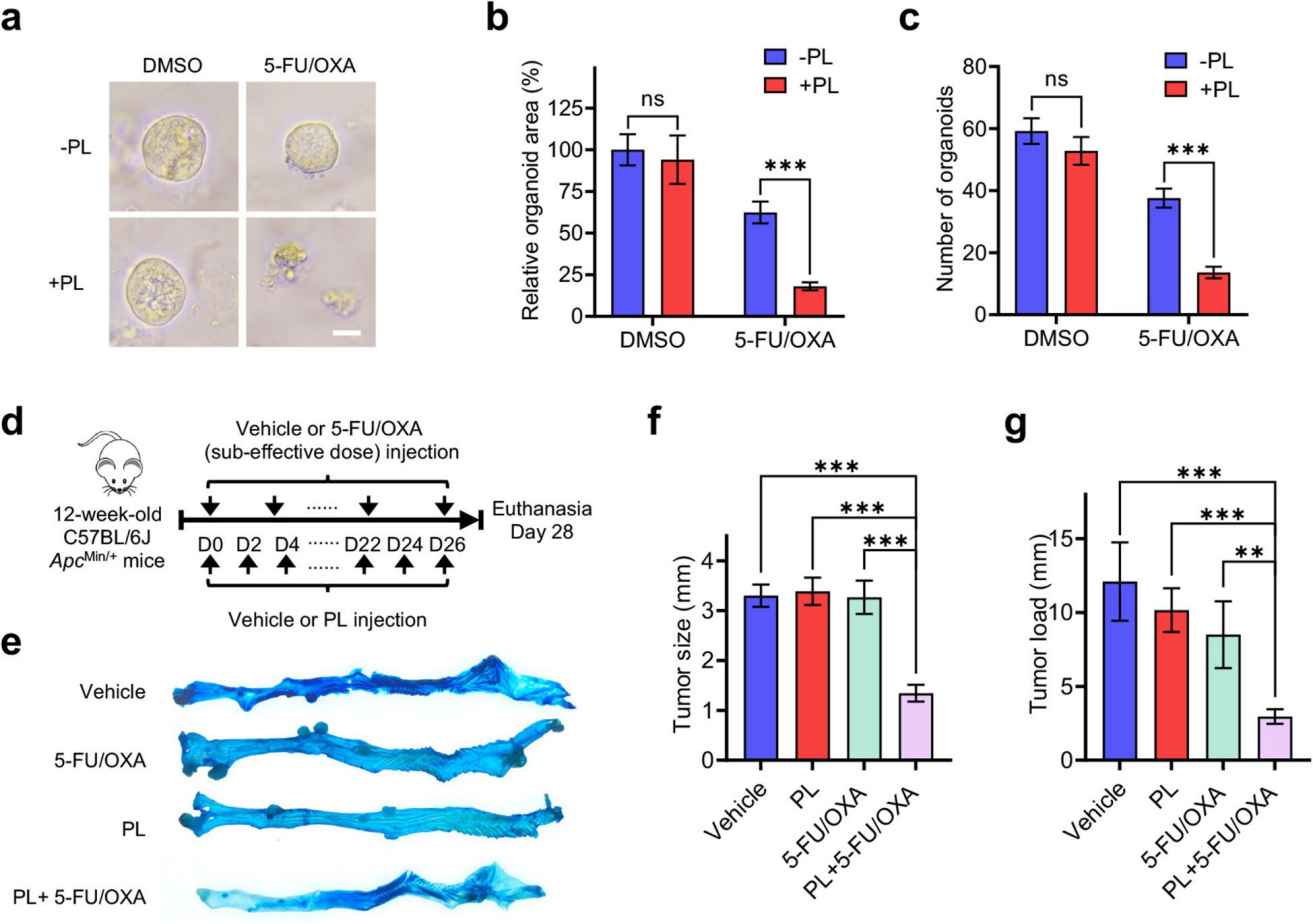
PL treatment sensitizes CRC with hyperactivated Wnt/β-catenin signaling to chemotherapeutic drugs ex vivo and in vivo. **a** Patient-derived organoids were treated with PL (2 µM) alone, chemotherapy alone (1 µM 5-FU and 1 µM OXA), or the combination of PL (2 µM) and chemotherapy (1 µM 5-FU and 1 µM OXA) for 5 days, with DMSO as a control. Representative images of patient-derived organoids in each group are shown. Scale bar, 50 µm. **b**, **c** Quantification of organoid size (**b**) and number (**c**) in each indicated group shown in (**a**). In (**b**), organoid size was normalized to the DMSO control group (set to 100%). Data were summarized from 10 individual organoids or 5 random fields in each indicated group. **d** Schematic illustrating the experimental timeline for the *Ap*c^min/+^ mouse model. **e** Representative images of methylene blue (MB)-stained colons from the indicated groups of *Ap*c^min/+^ mice. **f**, **g** Quantification of tumor size (**f**) and tumor load (**g**) in *Ap*c^min/+^ mice after intraperitoneal administration of PL (5 mg/kg) alone, a sub-effective dose of chemotherapy alone (2.5 mg/kg 5-FU and 2.5 mg/kg OXA), PL (5 mg/kg) plus chemotherapy (2.5 mg/kg 5-FU and 2.5 mg/kg OXA), or vehicle control. n = 5 mice per group. Statistical significance was determined by two-tailed Student’s t-test or one-way ANOVA; for tumor load, Kruskal–Wallis followed by Dunn’s test was employed. ns, *p* > 0.05; ***, *p* < 0.001

To further evaluate the impact of PL on CRC with aberrantly activated Wnt/β-catenin signaling, *Apc*-mutant patient-derived organoids and *Apc*^min/+^ mice, which harbor a mutated *Apc* tumor suppressor and spontaneously develop colon adenomas, were employed. Indeed, PL alone did not result in any obvious change in organoids, whereas chemotherapeutic drugs caused a moderate reduction in the number and size of the organoids (Fig. 6a-c). Interestingly, both the number and size of CRC organoids decreased dramatically when treated with the combination of PL and chemotherapeutic drugs compared with chemotherapeutic drugs alone (Fig. 6a-c). Twelve-week-old *Apc*^min/+^ mice (when colonic adenomas spontaneously developed) were treated with a sub-effective dose of chemotherapeutic drugs (2.5 mg/kg 5-FU and 2.5 mg/kg OXA) every four days, and PL was injected intraperitoneally every two days (Fig. 6d). Of note, the combination of PL and sub-effective chemotherapeutic drugs remarkably suppressed tumor growth compared with chemotherapy alone in *Apc*^min/+^ mice, as reflected by decreased tumor size and tumor load (Fig. 6e-g), although it did not significantly affect tumor number (Additional file 1: Fig. S15). These results indicate that PL enhances the efficacy of chemotherapeutic drugs in CRC with hyperactivated Wnt/β-catenin signaling.

## Discussion

Given that approximately 90% of CRC cases harbor mutations that result in the aberrant activation of Wnt/β-catenin signaling pathway [34], small-molecular inhibitors targeting Wnt/β-catenin signaling have exhibited promising therapeutic effects on CRC therapy in recent years [35–37]. However, there are currently no FDA-approved specific Wnt/β-catenin pathway inhibitors for cancer treatment [38]. The failure of inhibitors is mainly due to their toxicity caused by simultaneous downregulation of all Wnt/β-catenin target genes, which are essential for tissue homeostasis [13]. Therefore, targeting a specific single target gene of Wnt/β-catenin signaling may be reasonable for the treatment of Wnt/β-catenin-addicted CRC in terms of both safety and efficiency.

Wnt/β-catenin hyperactivation was shown to be essential for insensitivity to chemotherapy drugs in CRC cells [39, 40]; however, the underlying mechanism is still obscure. Here we report the identification of *TTI1* as a direct transcriptional target of Wnt/β-catenin signaling in CRC (Fig. 1h-m; Additional file 1: Fig. S16). TTI1 expression positively correlates with β-catenin, reflecting the activation of Wnt/β-catenin signaling at both the mRNA and protein levels. Gain-of-function experiments further supported this regulatory cascade, as ectopic β-catenin increased TTI1 expression together with ATM and ATR abundance, whereas ectopic TTI1 increased ATM and ATR without affecting β-catenin (Additional file 1: Fig. S17a-c). Notably, *NOB1* and *PHGDH* were also identified in the initial screening. However, unlike *TTI1*, neither gene showed a significant decrease upon β-catenin silencing in CRC cells (Additional file 1: Fig. S1a), and therefore TTI1 was prioritized for further investigation. Although *PHGDH* and *NOB1* have been implicated in tumor metabolism/proliferation in previous studies, their potential roles in Wnt/β-catenin-driven CRC chemoresistance remain to be defined. Many conventional chemotherapy drugs induce DNA damage, leading to the eventual apoptosis of cancer cells [41]. However, cancer cells often exhibit intrinsic biological mechanisms that activate various repair pathways, enabling them to repair DNA damage and evade chemotherapy-induced cell death [6, 7]. Of note, TTI1 expression markedly enhanced in CRC patients who did not respond to neoadjuvant chemotherapy compared with those who did respond (Fig. 1d-g). TTI1 deletion destabilizes ATM and ATR, the main regulators in DNA damage detection and the amplification of the DNA damage response. Although the precise mechanism remains to be defined, our data suggest that the loss of ATM/ATR upon TTI1 deficiency or PL treatment occurs through a proteasome-dependent process, as their mRNA levels were unchanged whereas MG132 partially restored their protein abundance. Given the established role of the TTT complex in PIKK maturation and stability, disruption of this complex could possibly impair proper ATM/ATR folding or maturation, which may in turn contribute to their proteasomal turnover; however, the specific E3 ubiquitin ligase(s) involved remain unknown. DSBs are among the most toxic and threatening forms of DNA damage [29], which are repaired by two predominant DNA repair pathways: HDR and NHEJ [31]. In addition, we detected DNA-PK in supplementary analyses (Additional file 1: Fig. S18), consistent with the established role of the TTT complex in maintaining the stability of PIKK family members. As a result, TTI1 knockout CRC cells exhibited increased sensitivity to chemotherapeutic drugs both in vitro and in subcutaneous xenograft mouse models. Consistently, PL alone had little effect on clonogenic growth in NCM460 cells, and its combination with 5-FU/OXA did not cause substantial additional growth inhibition under the same conditions (Additional file 1: Fig. S19), supporting the selective therapeutic potential of targeting the TTI1 axis.

Recent studies have demonstrated that RUVBL1/2 interact with TTT, a regulator of PIKK stability and assembly into a multiprotein complex [42, 43]. Thus, RUVBL1/2 have been considered ideal targets for regulating the functions of the TTT complex [23, 44]. As a novel inhibitor of RUVBL1/2, a natural small molecule, piperlongumine, binds to RUVBL1 and RUVBL2, then causes the dissociation of RUVBL1/2 from the TTT complex [23]. Concomitant with the dissociation, PL treatment reduced protein levels of TTI1, TTI2 and TELO2, but not RUVBL1 and RUVBL2, in CRC cells, thereby abolishing the function of TTT complex in stabilizing ATM and ATR proteins, leading to defective activation of DNA damage response signaling and impaired DNA repair (Fig. 5). Moreover, PL treatment acutely sensitizes CRC cells to chemotherapeutic drugs in vitro and in a subcutaneous xenograft mouse model, which is consistent with our observations in TTI1 deleted CRC cells (Fig. 5). The most frequent loss-of-function event in human CRC involves the *Apc* gene, causing aberrant activation of Wnt/β-catenin signaling. To further evaluate the effect of PL on chemotherapy in CRC with hyperactivated Wnt/β-catenin signaling, *Apc*-mutant patient-derived organoids and *Apc*^min/+^ mice were employed in our study. Of note, PL significantly improves the efficacy of chemotherapeutic drugs in both models of CRC with hyperactivated Wnt/β-catenin signaling (Fig. 6).

Despite these findings, several limitations remain to be addressed. First, while we demonstrated that TTI1 deficiency triggers the proteasomal turnover of ATM and ATR, the specific E3 ubiquitin ligase responsible for this process has not yet been identified. Second, as a central scaffold of the TTT complex, TTI1 also coordinates the stability of other PIKK family members, such as mTOR and DNA-PKcs; although our data emphasize the ATM/ATR-dependent DNA damage response, the potential roles of these other kinases in Wnt-driven resistance warrant further investigation. Finally, while piperlongumine was used to pharmacologically suppress the TTT complex and sensitize CRC cells to chemotherapy, it acts indirectly by targeting the RUVBL1/2-TTT interaction rather than TTI1 itself. The development of more potent and specific small-molecule inhibitors or PROTACs that directly target TTI1 will be crucial for the clinical translation of our findings to treat Wnt/β-catenin-activated colorectal cancer.

**Fig. 7:**
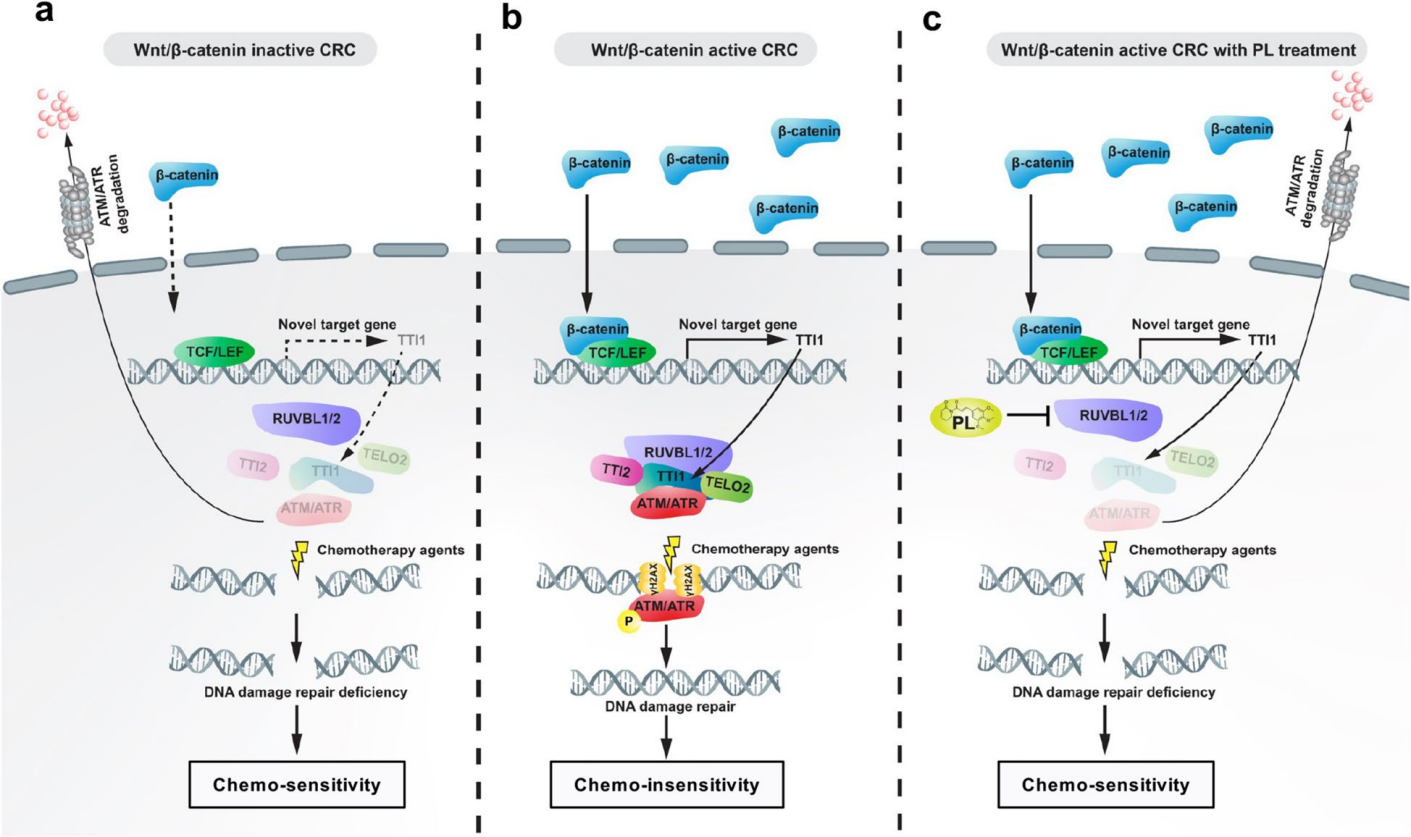
Schematic depiction of a role for TTI1 in controlling chemo-sensitivity of CRC by interlinking the Wnt/β-catenin, and DNA damage repair pathways. **a** In the inactive state of Wnt/β-catenin signaling, little β-catenin accumulates in the cytoplasm. The absence of nuclear β-catenin suppresses expression of TTI1, a novel Wnt/β-catenin target gene. Because TTI1 is required for stabilization of ATM and ATR, low TTI1 protein levels hamper formation of the TTT complex. Deficiency of ATM and ATR attenuates DNA damage repair and leads to hypersensitivity of CRC cells to chemotherapeutic drugs. **b** In the active state of Wnt/β-catenin signaling, excess β-catenin accumulates in the cytoplasm and translocates into the nucleus. There, β-catenin forms an active complex with the transcription factors TCF/LEF and enhances TTI1 expression. Together with TELO2 and TTI2, TTI1 forms an active TTT complex, which stabilizes ATM and ATR proteins with the assistance of RUVBL1/2. In response to chemotherapy-induced DNA damage, ATM and ATR phosphorylate DNA damage response transducers and effectors (e.g., H2AX, Chk1, and Chk2), thereby promoting efficient DNA repair and ultimately reducing sensitivity to chemotherapeutic drugs. **c** In Wnt/β-catenin-active CRC, PL treatment dissociates the TTT complex by directly binding to RUVBL1/2, resulting in destabilization of TTI1, TTI2, and TELO2. Subsequently, ATM and ATR are targeted for proteasomal degradation, which weakens DSB repair and sensitizes CRC cells to chemotherapeutic drugs

In conclusion, our findings reveal that TTI1 is a novel direct target of Wnt/β-catenin signaling in CRC cells. TTI1 is a central component of the TTT complex, which stabilizes ATM and ATR and thereby reduces sensitivity to chemotherapeutic drugs through orchestrating DNA damage responses (Fig. 7). TTI1 deletion, as well as PL treatment, which leads to significant TTI1 reduction, sensitizes CRC cells to the combination of 5-FU and OXA in vitro, ex vivo, and in vivo. Given that chemotherapeutic drugs are major DNA-damaging agents used in advanced and metastatic CRC, our findings highlight a potential strategy to selectively enhance the efficacy of chemotherapy in cancers with aberrant activated Wnt/β-catenin signaling.

## Methods

### Gene expression analysis

To find novel candidate downstream genes of β-catenin-dependent Wnt pathway, a comprehensive analysis of several transcriptomics datasets was performed. Transcriptome datasets, including GSE87429 and GSE69687, were obtained from the Gene Expression Omnibus (GEO) database, while TCGA-COAD data were retrieved from the UCSC XENA platform. Differentially expressed genes (DEGs) between si-β-catenin and si-NC HCT116 cell samples were screened from GSE87429 datasets [45]. Besides, DEGs between Wnt ligand secretion inhibitor (ETC159)-treated tumor xenografts and their vehicle control were screened from GSE69687 dataset [46]. Then, TCGA-COAD samples were categorized into high- and low-score groups based on the Wnt/β-catenin pathway score calculated using the gene set variation analysis (GSVA) algorithm [47], and differentially expressed genes (DEGs) between the two groups were identified. The DEGs between tumor and paraneoplastic tissues were explored in TCGA-COAD cohort. Finally, DEGs from various datasets were intersected to yield three candidate downstream genes of the Wnt/β-catenin pathway, namely TTI1, NOB1, and PHGDH (Fig. 1a).

### Patient sample collection

Patient samples were collected following the guidelines of the Declaration of Helsinki. Written informed consent was provided by all participants. The study received approval from the ethics committee of Xiangya Hospital, Central South University (Approval No. 202103507). This study was observational and did not involve an interventional clinical trial; therefore, clinical trial registration was not required.

### Cell culture and transfection

The HCT8, HCT116, and U2OS cell lines were obtained from the American Type Culture Collection. These cells were grown in DMEM (Servicebio, China) containing 10% Fetal Bovine Serum (Biological Industries, Beijing, China) and incubated at 37°C with a 5% CO_2_ atmosphere. Gene silencing was achieved through siRNA-mediated transfection of specific target genes using Lipofectamine 2000 reagent (Invitrogen; Thermo Fisher Scientific, USA), following the manufacturer's instructions. β-catenin and TTI1 siRNAs were designed and synthesized by GenePharma (Shanghai, China). The oligonucleotides sequences of the two independent siRNAs used for β-catenin and TTI1 knockdown are listed in Additional file 4: Table S3. And a lentiviral vector was used to stably transfer and express the exogenous EGFP and TTI1 fusion protein.

### Real-time quantitative PCR (RT-qPCR)

RNA was extracted using an RNA isolator (Vazyme, China) and converted to cDNA using cDNA Synthesis Kit (Novoprotein, China). RT-qPCR was performed with SYBR Green Supermix (Vazyme). The relative expression of target genes was quantified using the standard curve method and normalized to ACTB. The oligonucleotides information of the primers was listed in Additional file 4: Table S3.

### Immunoblotting

The cells were lysed using cytolysis buffer (50 mM Tris-HCl [pH 8.0], 50 mM NaCl, 0.5% Sodium deoxycholate, 1% NP-40, and a protease inhibitor cocktail). Cell extracts were separated by SDS-PAGE and subsequently probed with the relevant antibodies. The primary antibodies used in this study, along with their concentrations, are detailed in Additional file 5: Table S4.

### Dual-luciferase reporter gene

The Dual-Luciferase Reporter Assay System (Promega) was utilized to perform the dual-luciferase reporter assays. Genomic DNA from the HCT8 cell line was extracted according to the manufacturer's instructions (D1700-50, Solarbio, China). The TTI1 gene promoter region was amplified with the primers: F: GCCAGCCTGTTGTAGTCTGA and R: CGGAAGTGACGCGCTAAGTA. TTI1 promoter fragments were then subcloned into the firefly luciferase expression vector pTA-Luc (Clontech, USA). In addition, deletion mutants (-2000/-500 and -500/-1) and point mutants, including the binding site 1 mutation (B1-Mut), binding site 2 mutation (B2-Mut), and double mutations of both binding sites (Double-Mut), were generated for the TTI1 promoter-luciferase reporter vector. The Renilla luciferase plasmid served as a control. Cells were lysed 48 hours after transfection, and luciferase activity was measured using the Dual-Luciferase Assay Kit (Promega, USA).

### Chromatin immunoprecipitation (ChIP)

ChIP assays were performed as described [48]. A total of 1×107 HCT8 cells were cross-linked in 1.5 mM ethylene glycol-bis (succinimidyl succinate) (Thermo Scientific, USA) for protein-protein cross-linking, afterwards they were treated in 1% formaldehyde for at least 20 mins to preserve DNA-protein interactions. Chromatin was sheared by Qsonic Q800R (Qsonica, USA) for 6 min with the following setup: On 10 s, Off 30 s, intensity: 80%. Protein A/G magnetic beads and β-catenin antibodies (Santa Cruz sc-7199) were incubated with sonicated chromatin overnight at 4 °C, followed by washing and elution to isolate the enriched protein-DNA complexes. Following DNA purification, RT-qPCR was performed to quantify the TTI1 promoter. The primers used to amplify the TTI1 and GAPDH promoter regions (used as a reference control) were as follows: human TTI1 promoter (-262 to -92): 5’-AGGTCAAAGGGCAAGGGC-3’ and 5’-GCGGTCAAAGCTCTTTCCG-3’; human GAPDH promoter (-498 to -750): 5’- CTACAGGGCTGCAGGACATC-3’ and 5’- GTAGTGACACCGGACTGCTC-3’.

### Chromatin Fractionation

Cells were centrifuged at 3000 rpm for 5 minutes at 4 °C, resuspended in NETN buffer (20 mM Tris-HCl [pH 8.0], 100 mM NaCl, 1 mM EDTA, 0.5% NP-40, and a protease inhibitor cocktail), and incubated on ice for 20 minutes. After centrifugation at 3000 × g for 10 minutes, the supernatant (soluble fraction) was further centrifuged at 20,000 × g for 15 minutes. The pellets were washed with NETN buffer, resuspended in 0.2 M HCl, and sonicated for 10 seconds. After centrifugation at 20,000 × g for 15 minutes, the supernatant was neutralized and collected as the chromatin fraction.

### DNA pull-down assay

Biotin-labeled TTI1 promoter fragment and its mutants (B1-Mut, B2-Mut, Double-Mut) were obtained by PCR amplification using biotinylated primers. DNA pull-down assay was conducted according to the manufacturer’s instructions (Beaver Biosciences, China) [49]. Briefly, the streptavidin-coated magnetic beads (Beaver Biosciences) were prewashed twice with Buffer I (10 mM Tris-HCl pH 7.5, 1 M NaCl, 0.1% Tween-20, 1 mM EDTA). The biotinylated DNA fragments were then immobilized onto the beads by incubation in Buffer I at room temperature for 30 min. After removing the supernatant, the DNA-bead complexes were incubated with 100 μg cell lysates of HCT8 cells at 4 °C for 3 h (or overnight) to allow protein-DNA binding. The beads were subsequently washed three times with lysis buffer to remove non-specific binding. Finally, the enriched protein-DNA complexes were eluted, and the levels of β-catenin were analyzed by Western blotting.

### Generation of knockout cell lines using CRISPR/Cas9

Guide RNAs (sgRNAs, AGUUUUUUGAUACUCCUGAGG) were designed to knockout TTI1 with the online tool (https://design.synthego.com/#/). Based on the sgRNA sequence, forward primer CACCGAGTTTTTGATACTCCTGAGG and reverse primer AAACCCTCAGGAGTATCAAAAACTC were annealed to generate the DNA fragment, which was cloned into the pSpCas9(BB)-2A-GFP (PX458) (Addgene plasmid ID: 48138) as previously described [50]. The plasmid containing target gene-specific sgRNA was transfected in HCT8 and HCT116 cells, and those with green fluorescent were sorted using flow cytometry. The knockout of TTI1 was finally verified by WB assay.

### Neutral comet assays

DNA damage was assessed using the Comet Assay Kit (Trevigen). HCT8 WT and TTI1 KO cells were each mixed with molten agarose separately, placed onto slides, lysed, subjected to chromatin unwinding and neutral electrophoresis, and then stained for DNA visualization. Tail moments were quantified using CometScore software (Tri-Tek, USA) [51, 52].

### DSB repair reporter assay

The DSB repair reporter assay, including HDR and NHEJ efficiency, was performed by transfecting U2OS and HCT8 cells with TTI1-targeted or control siRNAs, followed by co-transfection with the HDR/NHEJ reporter and I-SceI to induce DSBs, and flow cytometry was used after 72 hours to measure GFP-positive cells, indicating DSB repair efficiency, as previously described [53, 54].

### Cell apoptosis assays

Cell apoptosis was evaluated using the Annexin V-FITC Apoptosis Detection Kit (Absin, China). Briefly, cells were collected and washed with cold PBS. Cell suspension was incubated with Annexin V-FITC for 10 min on ice in dark, then following incubated with propidium iodide for 5 min. Cells were analyzed on a DxP Athena™ Cytometers (Cytek Biosciences, USA), as described previously [55].

### Cell viability assay

Cell viability was evaluated after treating cells with the compounds at specified concentrations for 72 hours in 96-well plates. The MTT assay (Coolaber Science & Technology, China, SK2050) was used to determine cell viability following the manufacturer's protocol. Briefly, after the treatment period, cells were incubated with 10% MTT solution (5 mg/ml) for 4 hours. Subsequently, the cells were treated with a triplex solution (1:1) consisting of 0.012 M HCl, 5% isobutanol, and 10% SDS, and incubated for 12-16 hours before measuring absorbance at OD570 [56]. The survival rates of drug-treated cells were normalized to those of untreated controls for data visualization. Three independent experiments were performed.

### Mouse xenograft models

Five-week-old BALB/c nude mice were housed in a specific-pathogen-free environment. All animal procedures were approved by the Institutional Animal Care and Use Committee (IACUC) of Hunan SJA Laboratory Animal Co., Ltd. (Approval No. IACUC-SJA2022107-2). TTI1 WT or TTI1 KO HCT8 cells (2×10⁶) were resuspended in PBS injected subcutaneously into the hind flank. Tumor volume was measured bi-daily, and calculated with the formula V = length× width²×0.5. The maximum tumor diameter did not exceed 2.0 cm at any point, adhering to established animal welfare guidelines. All mice were euthanized 13 days post-injection for tumor harvesting and fixation in 4% paraformaldehyde.

### Apcmin/+ mice models

C57BL/6J Apcmin/+ mice, carrying a c.2549T>A (p.L850*) nonsense mutation, were described previously [57]. Ten- to twelve-week-old Apcmin/+ mice, maintained in a specific pathogen-free environment, were euthanized four weeks following the intraperitoneal administration of either the drug or vehicle control. The colon adenomas in these mice were visualized and quantified as previously outlined [58, 59]. In brief, mice were euthanized at approximately 16 weeks of age. The colon tissue was dissected, rinsed with cold PBS, and longitudinally opened, then fixed in 10% neutral buffered formalin overnight at 25 °C, and stained with 0.2% (w/v) methylene blue solution. The adenomas were quantified and size measured under a dissecting microscope. Tumor size and load per mouse were calculated by averaging the diameters of all tumors and summing their areas for each individual mouse.

### Immunohistology

After euthanasia, xenograft tumors were excised, rinsed with cold PBS, and fixed in 10% formalin for 24 hours. The tissues were embedded in paraffin, and 5 μm sections were cut for immunofluorescence. Following antigen retrieval, sections were incubated with primary antibodies overnight at 4 °C, followed by secondary antibody incubation for 2 hours at room temperature. Finally, the sections were stained with DAPI (Absin) and examined under a fluorescence microscope (Zeiss).

### Terminal deoxynucleotidyl transferase dUTP nick end labeling (TUNEL) assays

The TUNEL assay (Vazyme) was performed on dewaxed tumor sections to assess apoptosis. After proteinase K treatment and equilibration buffer incubation, the TUNEL reaction mixture was applied and incubated for 60 minutes at 37°C. The sections were then stained with DAPI and examined under a fluorescence microscope.

### Organoid culture

Colon adenocarcinoma tissue samples from a 75-year-old female patient (AJCC stage IIA) were collected at Xiangya Hospital, and processed to develop CRC organoids using established methods [60]. Tumor and normal tissues were rinsed, sliced into 1-3 mm³ pieces, and treated with Gentle Cell Dissociation Reagent (StemCell Technologies, Canada, 100-0485) and ROCK inhibitor (MCE, HY-10071). After filtration and centrifugation, the pellet was resuspended in 70% matrigel (Corning, 356231) and plated in 24-well plates. Organoid growth medium (StemCell Technologies, 06010) was added, supplemented with 1% Penicillin-Streptomycin and 15 mg/mL Gentamicin, was added to each well. The organoids were cultured at 37°C with 5% CO_2_, with medium changes every 2-3 days and passaged after 7-10 days.

### Treatment of organoids with siRNA or chemicals

Organoids in matrigel were washed with DPBS, disrupted by pipetting, and digested with Gentle Cell Dissociation Reagent for 30 minutes. After adding DMEM with 1% BSA, the organoids were resuspended, centrifuged, and transfected with 0.5 µL Lipo2000 for 4 hours. The cells were then resuspended in DMEM/F12, centrifuged, and plated in 70% matrigel. For PL/5-FU/OXA treatment, organoids were treated with vehicle, 2 µM PL, 1 µM OXA, or 5-FU after 3 days for 5-7 days. Organoid size was calculated using the formula Size = π × (d/2)² (where d is the diameter), and the number of organoids per well was counted after 8-10 days.

### Statistical analysis

Statistical analyses were performed using R software or GraphPad Prism. Data are presented as mean ± SEM. For comparisons between two groups, a two-tailed Student’s t-test (paired or unpaired) was used. For multiple group comparisons, one-way or two-way ANOVA followed by Tukey’s post-hoc test was applied for normally distributed data. For non-parametric data, including tumor numbers and loads in mouse models, the Kruskal-Wallis test followed by Dunn’s multiple comparisons test was uniformly employed. The significance was set at *p* < 0.05 (*), *p* < 0.01 (**), or *p* < 0.001 (***), with "ns" indicating no significance.

## Supplementary Information

Additional file 1.

Additional file 2.

Additional file 3.

Additional file 4.

Additional file 5.

## Data Availability

The transcriptome data used in this study are available on the UCSC XENA (https://xena.ucsc.edu/) and Gene Expression Omnibus database. The data generated in this study are available within the paper in the main text or the Supplementary file. Additional resources and data related to this paper can be requested from the corresponding authors. The public transcriptomic datasets analyzed in this study are available from the Gene Expression Omnibus (GEO) under accession numbers GSE87429 and GSE69687, and from the TCGA-COAD cohort through the UCSC XENA platform.
