# Supplementary material for: TELO2-interacting protein 1 (TTI1), a novel Wnt/β-catenin target gene, decreases chemo-sensitivity in colorectal cancer by modulating DNA damage responses
Source: Mol Biomed. 2026 Jun 12;7:88. doi: 10.1186/s43556-026-00475-8 (PMC13263369; doi:10.1186/s43556-026-00475-8)
Supplement: Supplementary file 1 — Additional file 1. [file 43556_2026_475_MOESM1_ESM.pdf]

# Supplementary Figures

## TELO2-interacting protein 1 (TTI1), a novel Wnt/β-catenin target gene, decreases chemo-sensitivity in colorectal cancer by modulating DNA damage responses

*Yuqiao Chen*[1,2]†, *Zheng Chen*[1,3]†, *Ya Wang*[1,4], *Yuanbing Yao*[1], *Youyu Zhang*[2], *Wentao Huang*[2], *Kun Song*[5], *Fengbo Tan*[5], *Fei Long*[6], *Changwei Lin*[6], *Qian Zhang*[7], *Wei Zhu*[8], *Wei Zhuang*[2], *Jianhua Zhou*[8], *Heli Liu*[5], *Shuai Xiao*[4], *and Kai Fu*[1,9,10,11]*

[1] Institute of Molecular Precision Medicine and Hunan Key Laboratory of Molecular Precision Medicine, Department of General Surgery, Xiangya Hospital, Central South University, Changsha, Hunan, China

[2] Department of Thoracic Surgery, Xiangya Hospital, Central South University, Changsha, Hunan, China

[3] Department of Neurosurgery, Xiangya Hospital, Central South University, Changsha, Hunan, China

[4] The First Affiliated Hospital, Cancer Research Institute, Hengyang Medical School, University of South China, Hengyang, Hunan, China

[5] Department of Gastrointestinal Surgery, Xiangya Hospital, Central South University, Changsha, Hunan, China

[6] Department of Gastrointestinal Surgery, The Third Xiangya Hospital, Central South University, Changsha, Hunan, China

[7] Department of Nutrition and Health, China Agricultural University, Beijing, China

[8] Department of Pathology, Xiangya Hospital, Central South University, Changsha, China

[9] MOE Key Lab of Rare Pediatric Diseases & Hunan Key Laboratory of Medical Genetics of the School of Life Sciences, Central South University, Changsha, Hunan, China

[10] National Clinical Research Center for Geriatric Disorders, Changsha, Hunan, China

[11] Hunan Key Laboratory of Aging Biology, Xiangya Hospital, Central South University, Changsha, Hunan, China

†These authors contributed equally to this work.

**Corresponding Author:**

Kai Fu

E-mail: fu_kai@csu.edu.cn

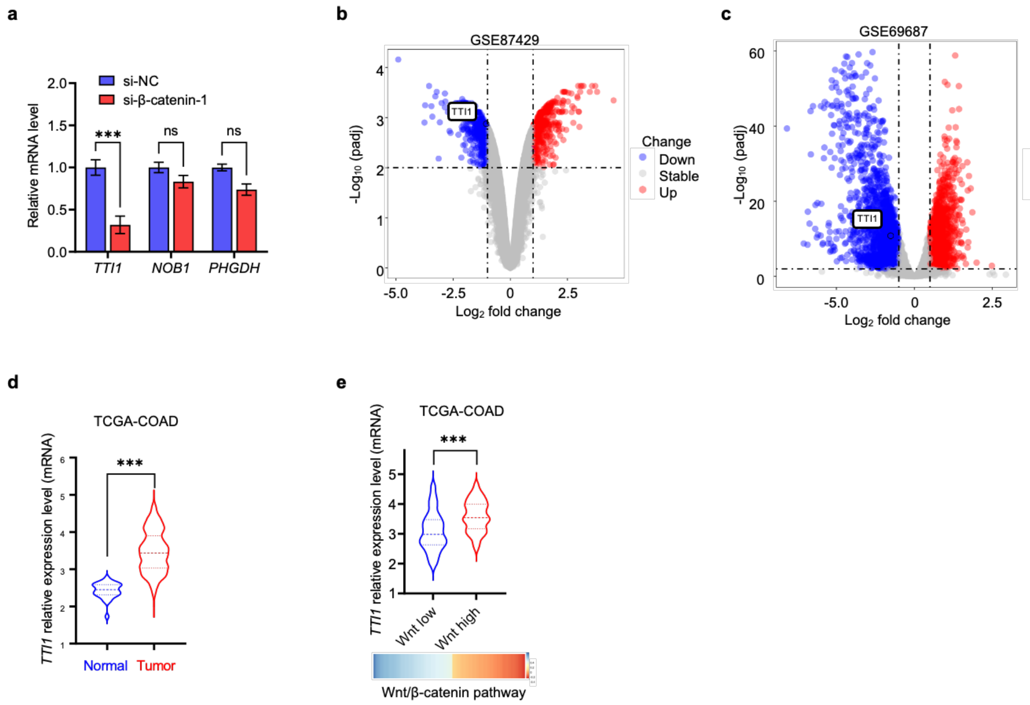


**Additional file 1: Fig. S1. A comprehensive bioinformatic analysis for screening novel downstream genes of Wnt/β-catenin pathway.** (a) HCT8 cells were transfected with si-NC or si-β-catenin-1 for 72 h. The mRNA levels of indicated genes were measured by RT-qPCR. (b) The volcano plot depicted the differentially expressed genes (DEGs) between si-β-catenin-1 and si-NC of HCT116 cells using the microarray data of GSE87429. The threshold values ($Log_2$(Fold Change) > 1 and adjust-$p < 0.01$) are indicated by dashed lines. The blue dots represent downregulated genes, and the red dots represent upregulated genes. (c) The volcano plot depicted DEGs between Wnt ligand secretion inhibitor (ETC159)-treated patient-derived xenografts and control from GSE69687 dataset. (d) The violin plot demonstrated the expression of TTI1 in tumor and paraneoplastic tissues in the TCGA-COAD cohort. (e) Based on Wnt/β-catenin pathway score calculated by the gene set variation analysis (GSVA) algorithm, TCGA COAD samples were divided into high- and low-score groups, and the violin plot showed the expression of TTI1 between the two groups.

**Additional file 1: Fig. S2**

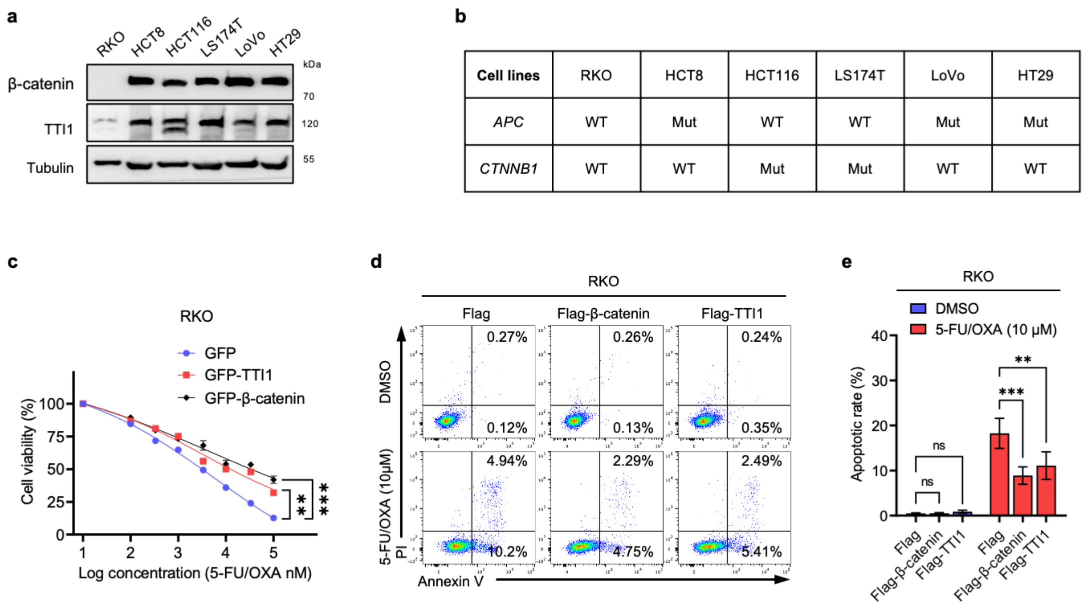


**Additional file 1: Fig. S2. Overexpression of β-catenin and TTI1 decreases the efficacy of chemotherapy drugs in colorectal cancer cells with intact Wnt/β-catenin pathway.** (a) The whole cell lysates from different CRC cell lines, including RKO, HCT8, HCT116, LS174T, LoVo and HT29, were collected and immunoblotted for indicated protein. (b) Mutational status of *APC* and *CTNNB1* genes in CRC cell lines used in (a). (c) RKO cells were transduced with lentiviral vectors carrying GFP, GFP-β-catenin, or GFP-TTI1 plasmids. These cells were treated with chemotherapeutic agents at various concentrations for another 48 h, and cell viability was determined using MTT assay. (d) RKO cells transfected with indicated plasmids were treated with DMSO or chemotherapeutic agents (10 μM 5-FU and 10 μM OXA) for 24 h, followed by PI/Annexin V staining and flow cytometry analysis. (e) Quantification of apoptotic cell rate in (d). Statistical significance was determined by one-way ANOVA. ns, $p > 0.05$; **, $p < 0.01$; ***, $p < 0.001$.

**Additional file 1: Fig. S3**

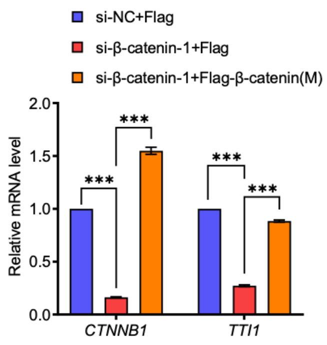


**Additional file 1: Fig. S3. The mRNA levels of indicated genes in HCT8 cells after sequential transfection with siRNAs and plasmids.** HCT8 cells were transfected with the indicated siRNAs for 36 h and then transfected with the corresponding plasmids for another 36 h. The mRNA levels of the indicated genes were measured by RT-qPCR. Data are presented as the mean ± SEM of three independent experiments. Statistical significance was determined by one-way ANOVA. ***, $p < 0.001$.

**Additional file 1: Fig. S4**

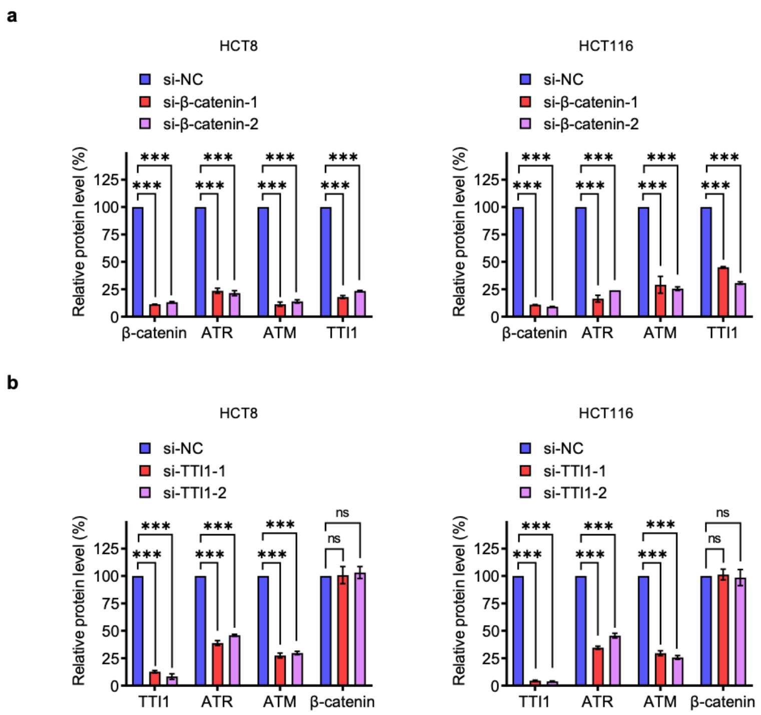


**Additional file 1: Fig. S4. Quantification of the protein levels in β-catenin- or TTI1-depleted CRC cells.** (a) HCT8 (left) and HCT116 (right) cells were transfected with si-NC or two independent β-catenin-targeting siRNAs for 72 h. The relative protein levels of β-catenin, ATR, ATM and TTI1 were quantified from the immunoblotting results shown in Fig. 2b. (b) HCT8 (left) and HCT116 (right) cells were transfected with si-NC or two independent TTI1-targeting siRNAs for 72 h. The relative protein levels of TTI1, ATR, ATM and β-catenin were quantified from the immunoblotting results shown in Fig. 2d. Data are presented as the mean ± SEM of three independent experiments. Statistical significance was determined by one-way ANOVA. ns, $p > 0.05$; ***, $p < 0.001$.

**Additional file 1: Fig. S5**

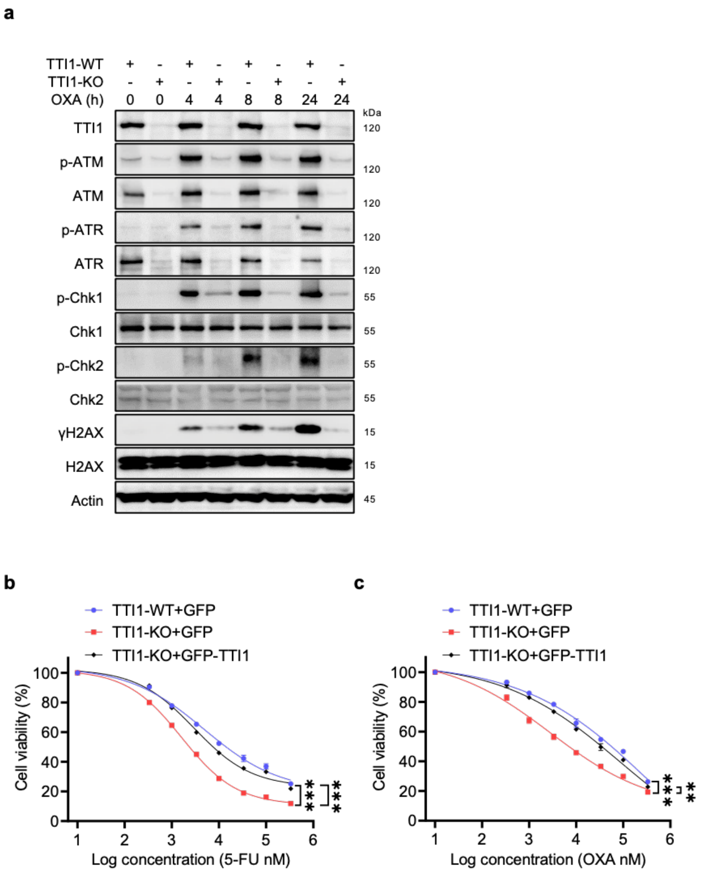


**Additional file 1: Fig. S5. Chemotherapeutic drugs-triggered DNA damage repair signaling cascade was substantially reduced in TTI1 depleted CRC cells.** (a) The whole cell lysates of TTI1-WT and TTI1-KO HCT8 cells were derived at indicated time points post treatment of 10 µM oxaliplatin (OXA) and immunoblotted for indicated protein. (b-c) The cell viability assay revealed the survival fraction of TTI1-WT and TTI1-KO HCT8 cells expressing GFP or GFP-TTI1 72 h post treatment with indicated concentration of 5-FU (b) or OXA (c).

**Additional file 1: Fig. S6**

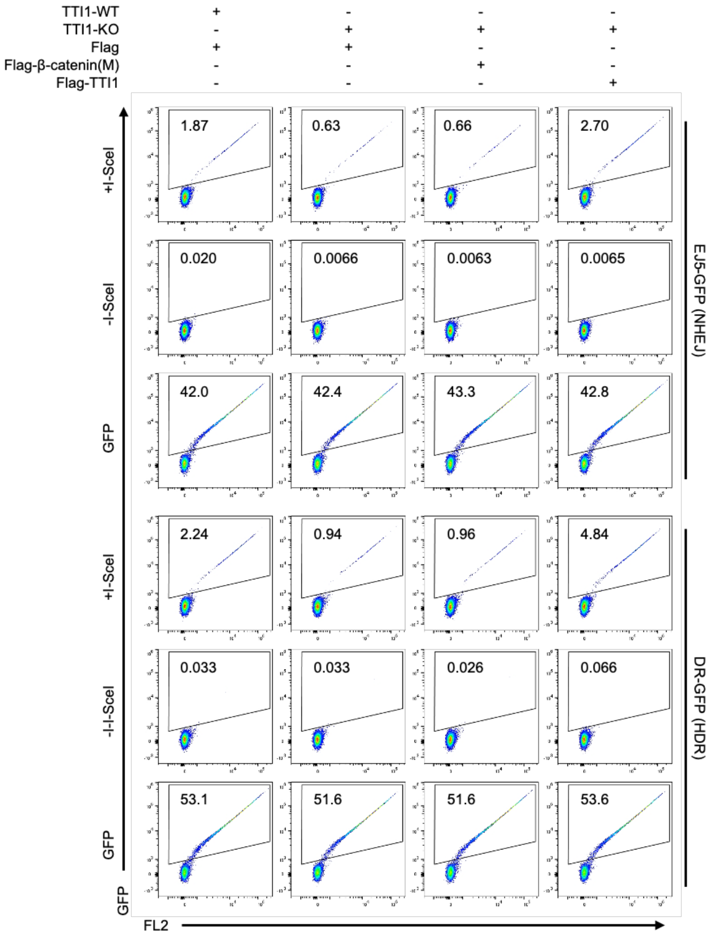


**Additional file 1: Fig. S6. Representative flow cytometry analyses showing HDR and NHEJ repair efficiency in TTI1-deficient HCT8 cells after re-expression of TTI1 or β-catenin(M).** TTI1-WT and TTI1-KO HCT8 cells were first transfected with empty vector (Flag), siRNA-resistant Flag-β-catenin(M), or Flag-TTI1 as indicated. 24 hours later, cells were co-transfected with the HDR reporter (DR-GFP) or NHEJ reporter (EJ5-GFP) together with I-SceI to induce DSBs at unique I-SceI sites. 48 hours after reporter transfection, GFP-positive cells were analyzed by flow cytometry to determine HDR or NHEJ repair efficiency.

**Additional file 1: Fig. S7**

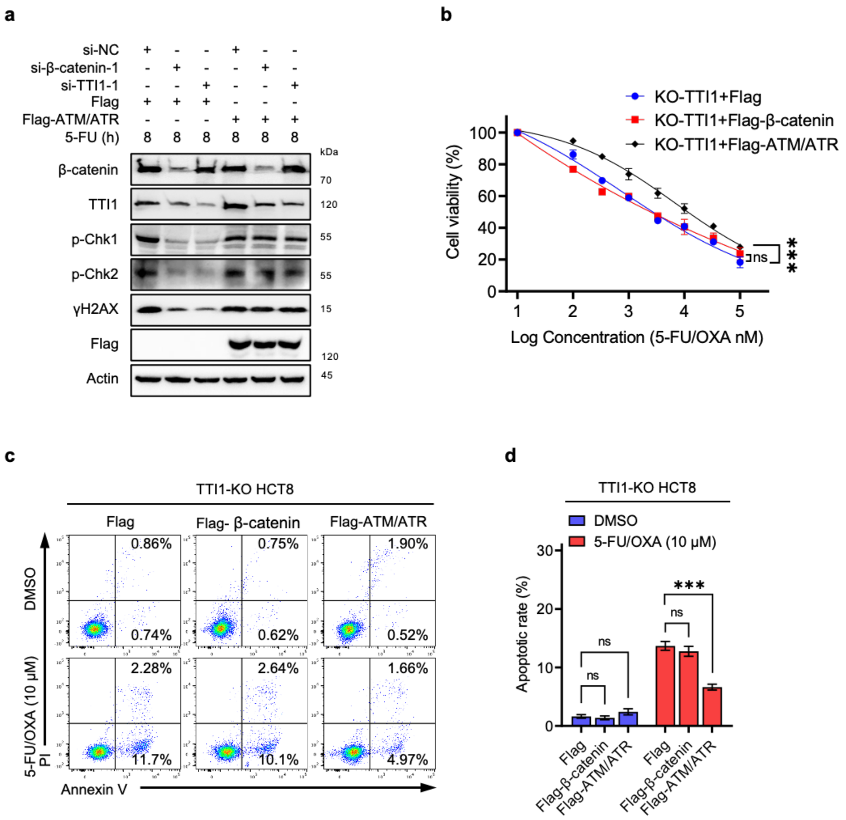


**Additional file 1: Fig. S7. Ectopic expression of ATM and ATR restores DNA damage-induced signaling activation and reduces the sensitivity of TTI1-deficient cells to chemotherapy drugs.** (a) After knockdown of β-catenin or TTI1 for 24 h, HCT8 cells were transfected with Flag or Flag-ATM/ATR plasmids for 48 h. The cells were treated with 10 µM 5-FU for 8 h, then whole cell lysates were derived and immunoblotted for indicated proteins. (b) TTI1-KO HCT8 cells were transfected with Flag, Flag-β-catenin, or Flag-ATM/ATR. 48 h later, the cells were treated with chemotherapeutic agents at various concentrations for another 48 h, and cell viability was determined using MTT assay. (c) TTI1-KO HCT8 cells were transfected with indicated plasmids. 48 h later, the cells were treated with DMSO or chemotherapeutic agents (10 µM 5-FU and 10 µM OXA) for 24 h, followed by PI/Annexin V staining and flow cytometry analysis. (d) Quantification of apoptotic cell rate in (c). Statistical significance was determined by one-way ANOVA. ns, $p > 0.05$; ***, $p < 0.001$.

**Additional file 1: Fig. S8**

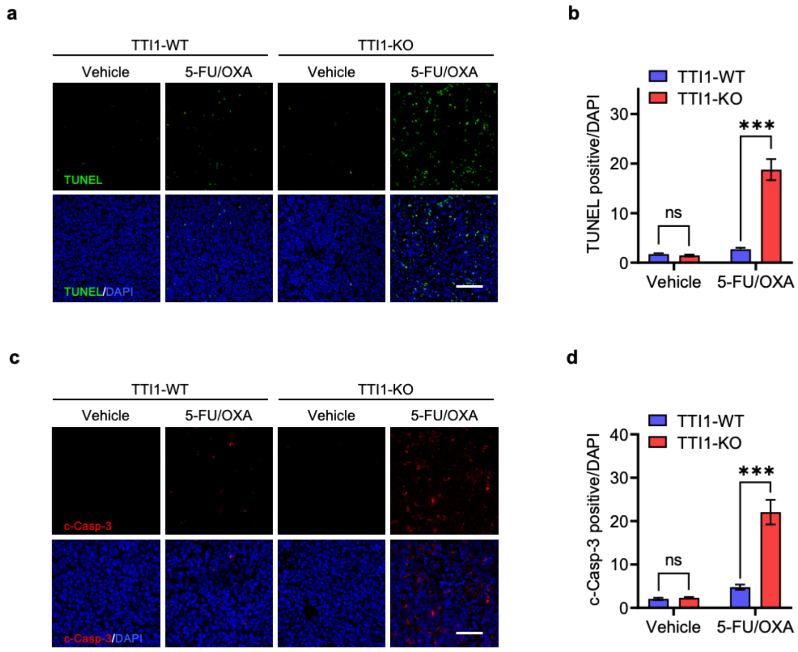


**Additional file 1: Fig. S8. Deletion of TTI1 sensitizes CRC cells to chemotherapeutic drugs-induced apoptosis in xenograft mouse model.** (a) The apoptotic signals were detected by TUNEL staining of the tumor sections. Apoptotic cells were visualized by TUNEL (TdT, green), and the nucleus was stained with DAPI. Scale bar, 100 μm. (b) Relative TUNEL staining from 5-10 random fields, as in (a), were quantified. (c) Representative immunofluorescence micrographs of cleaved-Caspase-3 (c-Casp-3) in the tumor sections. Scale bar, 100 μm. (d) Quantification of the percentage of cleaved-Caspase-3 positive in the tumor sections, as in (c). Data were summarized from 5-10 random fields. Statistical significance was determined by two-tailed Student's t-test. ns, $p > 0.05$; ***, $p < 0.001$.

**Additional file 1: Fig. S9**

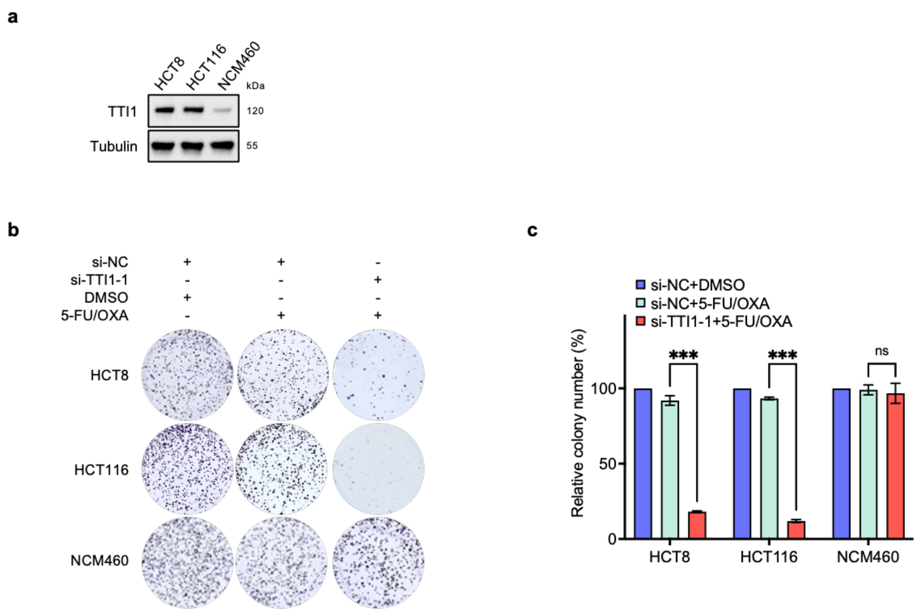


**Additional file 1: Fig. S9. TTI1 depletion selectively enhances the inhibitory effect of 5-FU/OXA on clonogenic survival in CRC cells, but not in normal colonic epithelial cells.** (a) Whole-cell lysates from HCT8, HCT116 and NCM460 cells were subjected to immunoblotting for TTI1, with Tubulin as a loading control. (b) HCT8, HCT116 and NCM460 cells were transfected with si-NC or si-TTI1. After 48 h, cells were exposed to DMSO or 5-FU/OXA (20 μM) for 24 h and then cultured in drug-free medium. Colony formation was assessed 8 days later. Representative colony formation images are shown. (c) Quantification of colony numbers in (b). Data are presented as the mean ± SEM of three independent experiments. Statistical significance was determined by one-way ANOVA. ***, $p < 0.001$.

**Additional file 1: Fig. S10**

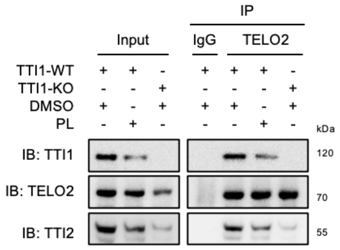


**Additional file 1: Fig. S10. PL disrupts the association of TTI1 with the TTT complex in HCT8 cells.** HCT8 TTI1-WT and TTI1-KO cells were treated with DMSO or PL (2 μM) for 12 h. Whole-cell lysates were subjected to immunoprecipitation with an anti-TELO2 antibody or control IgG and then immunoblotted for TTI1, TELO2 and TTI2. Input samples are shown on the left.

**Additional file 1: Fig. S11**

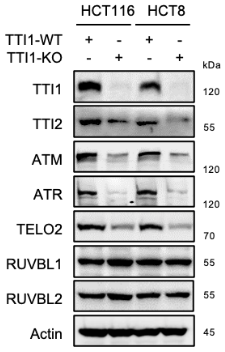


**Additional file 1: Fig. S11. TTI1 deletion reduced protein levels of all TTT components and ATM/ATR, but not RUVBL1/2.** The levels of indicated proteins were examined by immunoblotting in TTI1-WT and TTI1-KO HCT8/HCT116 cells.

**Additional file 1: Fig. S12**

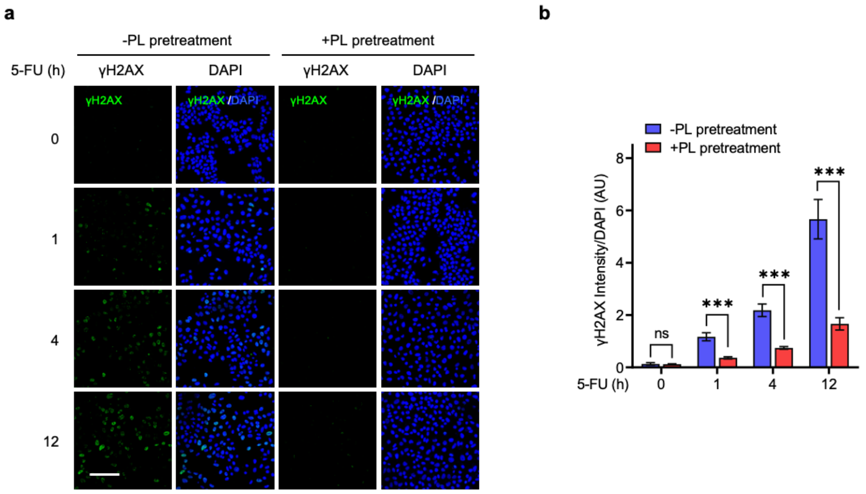


**Additional file 1: Fig. S12. Chemotherapeutic drugs-triggered γH2AX accumulation was severely attenuated in PL pretreated CRC cells.** (a) Representative immunofluorescence micrographs of γH2AX in HCT8 pretreated with or without 2 μM PL pretreatment were collected at the indicated time points following 10 μM 5-FU treatment, with nuclei counterstained by DAPI. Scale bar, 100 μm. (b) Quantitative analysis of γH2AX foci grayscale values per cell in (a). 5-10 random fields of view were counted at each time point. Statistical significance was determined by two-tailed Student's t-test. ns, $p > 0.05$; ***, $p < 0.001$.

**Additional file 1: Fig. S13**

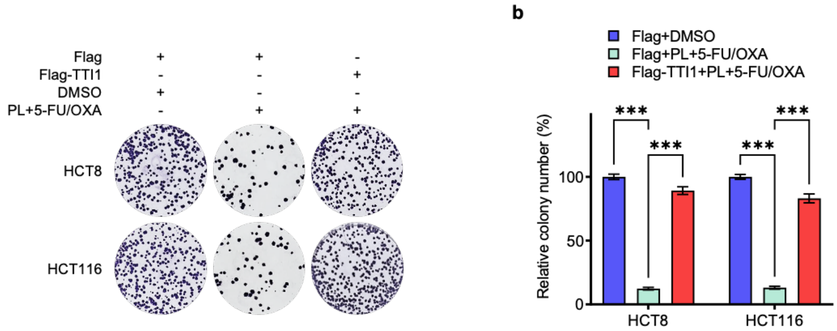


**Additional file 1: Fig. S13. Re-expression of TTI1 attenuates the inhibitory effect of PL plus 5-FU/OXA on clonogenic survival in CRC cells.** (a) HCT8 and HCT116 cells were transfected with the Flag vector control or Flag-TTI1 plasmid. After 24 h, cells were pretreated with PL (2 μM) for 12 h and then exposed to PL plus 5-FU/OXA (20 μM) for another 24 h, followed by drug withdrawal. Colony formation was assessed 8 days later. (b) Quantification of colony numbers in (a). Data are presented as the mean ± SEM of three independent experiments. Statistical significance was determined by one-way ANOVA. ***, $p < 0.001$.

**Additional file 1: Fig. S14**

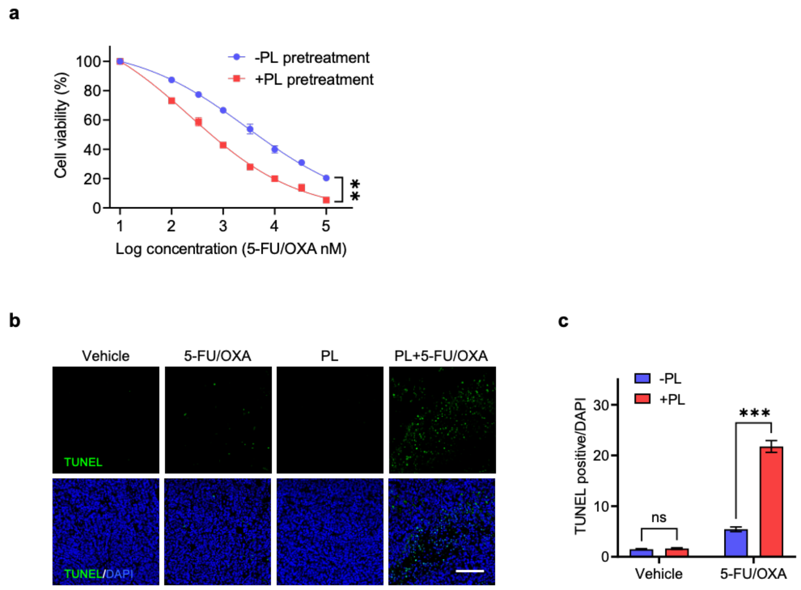


**Additional file 1: Fig. S14. PL treatment sensitizes CRC cells to chemotherapy.** (a) The cell viability assay revealed the survival fraction of HCT8 cells, which were pretreated with or without PL (2 μM) for 8 hours, and following the treatment of the indicated dose of chemotherapeutic agents (5-FU and OXA) for 72 h. (b) TUNEL labeling of tumor slices was used to determine the apoptosis in each group. Scale bar, 200 μm. (c) Relative TUNEL staining from 5-10 random fields, as in (b), were quantified. Statistical significance was determined by two-tailed Student's t-test. ns, $p > 0.05$; ***, $p < 0.001$.

**Additional file 1: Fig. S15**

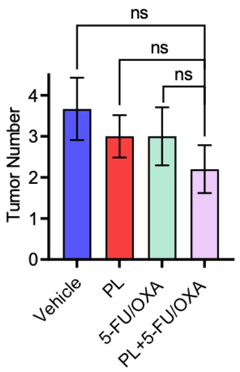


**Additional file 1: Fig. S15. Quantification of tumor number in the colons from *Apc*$^{min/+}$ mice following MB staining.** The mice were intraperitoneally injected with PL (5 mg/kg), chemotherapy (2.5 mg/kg 5-FU and 2.5 mg/kg OXA), a combination of PL (5 mg/kg) and chemotherapy (2.5 mg/kg 5-FU and 2.5 mg/kg OXA), or vehicle control. n=5 mice per group. Statistical significance of tumor number was calculated via Kruskal-Wallis followed by Dunn's test. ns, $p > 0.05$.

**Additional file 1: Fig. S16**

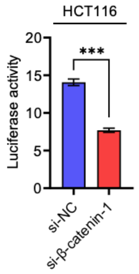


**Additional file 1: Fig. S16. The *TTI1* promoter activity reduced in β-catenin depleted HCT116 cells.** Luciferase activity of indicated *TTI1* promoter (-2000/-1) constructs was measured in HCT116 cells transfected with si-NC or si-β-catenin. pRL-TK renilla luciferase reporter construct was co-transfected to each sample to normalize transfection efficiency. The ratio of reporter luciferase activity to control renilla luciferase activity is indicated. Statistical significance was determined by two-tailed Student's t-test or one-way ANOVA. ns, $p > 0.05$; ***, $p < 0.001$.

**Additional file 1: Fig. S17**

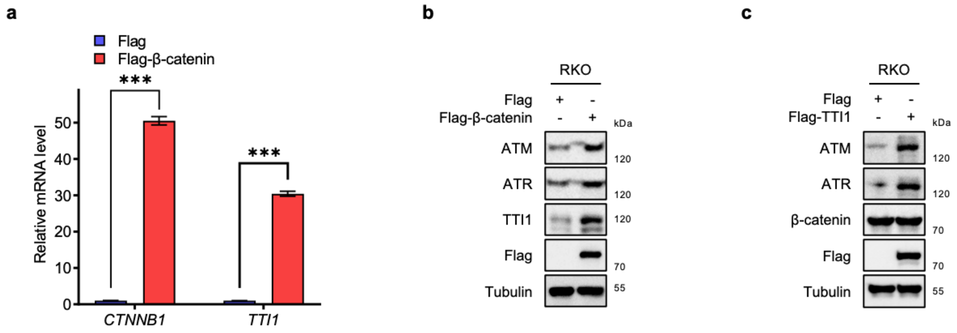


**Additional file 1: Fig. S17. Ectopic expression of β-catenin or TTI1 increases ATM and ATR protein levels in RKO cells.** (a-b) RKO cells were transfected with the Flag vector control or Flag-β-catenin plasmid for 36 h. The mRNA levels of β-catenin and TTI1 were measured by RT-qPCR (a), and whole-cell lysates were subjected to immunoblotting for ATM, ATR, TTI1 and Flag, with Tubulin as a loading control (b). (c) RKO cells were transfected with the Flag vector control or Flag-TTI1 plasmid for 36 h. Whole-cell lysates were subjected to immunoblotting for ATM, ATR, β-catenin and Flag, with Tubulin as a loading control. Data are presented as the mean ± SEM of three independent experiments. Statistical significance was determined by two-tailed Student's t-test. ***, $p < 0.001$.

**Additional file 1: Fig. S18**

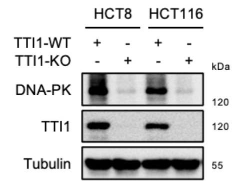


**Additional file 1: Fig. S18. Immunoblot analysis of DNA-PK expression in TTI1-WT and TTI1-KO CRC cells.** Whole-cell lysates from HCT8 and HCT116 TTI1-WT and TTI1-KO cells were subjected to immunoblotting for DNA-PK and TTI1, with Tubulin as a loading control.

**Additional file 1: Fig. S19**

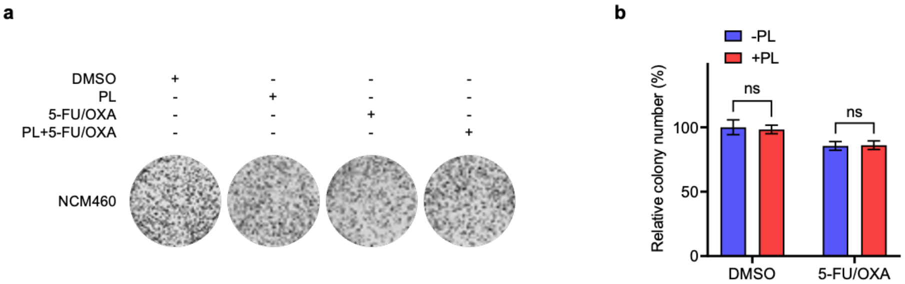


**Additional file 1: Fig. S19. PL pretreatment had little effect on the clonogenic growth of NCM460 cells, either alone or in combination with 5-FU/OXA.** (a) NCM460 cells were treated with DMSO, PL (2 μM), 5-FU/OXA (40 μM), or pretreated with PL (2 μM) for 12 h followed by co-treatment with PL (2 μM) and 5-FU/OXA (40 μM) for 24 h. After drug withdrawal, cells were cultured for 8 days and subjected to colony formation assay. (b) Quantification of colony numbers in (a). Data are presented as the mean ± SEM of three independent experiments. Statistical significance was determined by two-tailed Student's t-test. ns, $p > 0.05$.

**Additional file 2: Table S1.** The main clinical characteristics of CRC patients, whose tumor and normal specimens were used for immunohistochemistry and RT-qPCR in this study.

**Additional file 3: Table S2.** The main clinical characteristics of CRC patients with different chemotherapy sensitivity.

**Additional file 4: Table S3.** The oligonucleotides information of small interference RNA (si-RNA) and primer sequence of target genes.

**Additional file 5: Table S4.** The antibodies and their working concentration used in this study.
